# Social frailty in community-dwelling older adults: a scoping review

**DOI:** 10.1186/s12877-025-05971-0

**Published:** 2025-05-13

**Authors:** Cuiting Huang, Wachiranun Sirikul, Nida Buawangpong

**Affiliations:** 1 Department of Community Medicine, Faculty of Medicine, Chiang Mai University, Chiang Mai, 50200 Thailand; 2 Department of Nephrology, Affiliated Hospital of Youjiang Medical University for Nationalities, Baise, 533000 Guangxi Zhuang Autonomous Region China; 3 Department of Biomedical Informatics and Clinical Epidemiology, Faculty of Medicine, Chiang Mai University, Chiang Mai, 50200 Thailand; 4 Department of Family Medicine, Faculty of Medicine, Chiang Mai University, Chiang Mai, 50200 Thailand

**Keywords:** Social frailty, Community-dwelling older adults, Scoping review, Measurements, Health outcomes

## Abstract

**Background:**

The rising global population of older adults has increased attention on social frailty, significantly affecting physical, cognitive, and mental health.

**Objectives:**

This review systematically examines social frailty in community-dwelling older adults by exploring theoretical frameworks, measurement tools, determinants, health outcomes, and potential interventions.

**Methods:**

A scoping review following PRISMA-ScR guidelines was conducted with searches across PubMed, Embase, and CINAHL databases, covering studies up to November 4, 2024. Of the 2371 articles, 42 were included in the synthesis. The review focused on community-dwelling older adults aged 60 and above, aiming to understand the role of social frailty in this population’s health and well-being.

**Results:**

The findings revealed that social frailty is a complex, multidimensional phenomenon determined by physical function decline, cognitive impairment, and depression. It is also linked to adverse outcomes, including increased risks of cognitive decline, depression, disabilities, and mortality. The growing evidence underscores the need for targeted interventions to disrupt the cycle of worsening frailty. The variability in existing measurement tools, limiting their broad applicability, suggested a need for standardization and cross-cultural validation. We identified potential intervention strategies, including multicomponent physical exercise, social networking enhancement, and digital health interventions, but only one RCT specifically examined a robotic pet intervention targeting social frailty, and its findings were not significant.

**Conclusion:**

Social frailty measurement tools are based on two primary frameworks: one grounded in the Deficit Accumulation Model, suited for secondary data analysis and prospective or periodic screening, and the other based on the Social Needs Fulfillment Theory, which uses brief-item tools but requires validation in specific contexts. Social frailty is associated with increased morbidity and mortality among community-dwelling older adults. The studies employed physical workouts, social networking, and digital health interventions, showed a significant improvement in social engagement. However, none specifically aimed at reducing social frailty, underscoring the urgent need for the specific interventions and management for social frailty.

**Supplementary Information:**

The online version contains supplementary material available at 10.1186/s12877-025-05971-0.

## Introduction

As the global population ages, the proportion of older adults is projected to reach 18.1% by 2060 [1], creating significant challenges for health and social care systems. With an increasing focus on promoting healthy aging, social frailty has emerged as a critical concept. The concept of social frailty emerged as a distinct domain within frailty research when Gobbens et al. [2] proposed an integrated model differentiating physical, psychological, and social frailty. This model emphasized that social frailty was independent of physical or cognitive decline, establishing social frailty as a critical aspect of aging research. Social frailty refers to a lack of social support, connectedness, resources, and the fulfillment of basic needs [3]. It encompasses aspects such as social activities, social networks, and whether individuals live alone [4].​The prevalence of social frailty varies widely and is estimated to affect between 7.7% and 47% of older adults in different settings [5–8].

Social frailty is associated with other related concepts, such as loneliness, social isolation, social vulnerability (SV), and resilience, highlighting the need for a holistic approach to understanding it [9–11]. Loneliness refers to the subjective distress experienced when there is a perceived gap between desired and actual social relationships [12], whereas social isolation is an objective state characterized by a lack of social contacts or interactions [13]. In contrast, SV is a broader construct, encompassing an individual’s susceptibility to adverse health and social outcomes due to limited social resources, reduced participation, and environmental constraints [14]. Social frailty, however, specifically reflects a decline in social functioning, impairing an individual’s ability to maintain independence and overall well-being. This condition increases vulnerability to adverse health outcomes, including cognitive decline, permanent disability, and mortality [15]. Social frailty thus represents a substantial challenge for the care of older adults and the healthcare system overall. Despite its importance, there remains a limited understanding of social frailty, including its conceptual frameworks, effective assessment tools, and potential interventions.

Existing knowledge syntheses largely focus on the clinical aspects of frailty or only on aspects such as social isolation and loneliness, leaving social frailty underexplored. Furthermore, multiple definitions, frameworks, and tools were employed across studies, leading to fragmentation within the field [3, 16–18]. Current reviews [19, 20] have not clarified how conceptual frameworks influence assessment tools, the links between influencing factors and adverse outcomes, and which interventions may effectively address social frailty. Therefore, a comprehensive synthesis of the existing evidence is needed to understand social frailty better and to inform policy and practice in supporting healthy aging and improving the quality of life for older adults.

To address these gaps systematically and consider the emerging and multidimensional nature of social frailty, a scoping review was selected to map the current evidence comprehensively. This method enables a systematic examination of existing frameworks, determinants, and interventions while identifying gaps in the literature to guide future research priorities. This scoping review aimed to map the current landscape of research on social frailty among community-dwelling older adults, focusing on five key aspects: (1) Theoretical frameworks that underpin social frailty, (2) Measurement tools used to assess social frailty, (3)Determinants contributing to social frailty, (4) Health outcomes associated with social frailty, (5)Interventions and strategies designed to mitigate social frailty and promote healthy aging.

By synthesizing current research, this review offered a comprehensive overview of the conceptual, methodological, and practical aspects of social frailty, providing insights for future research, policy development, and healthcare interventions.

## Methods

This scoping review followed the PRISMA-ScR (Preferred Reporting Items for Systematic Reviews and Meta-Analyses Extension for Scoping Reviews) [21] guidelines to ensure a transparent and rigorous reporting process. The review protocol was developed before the search. It was guided by five primary objectives: mapping theoretical frameworks, measurement tools, determinants, health outcomes, and interventions related to social frailty among community-dwelling older adults.

### Eligibility criteria

In this scoping review, “community-dwelling older adults” referred to individuals aged 60 and above living independently or with family in non-institutional settings, such as private homes, rather than nursing homes or long-term care facilities. Only studies that explored the concept of social frailty, assessment tools, its determinants, and related health outcomes-including disability and mortality, as well as impairments in the physical (e.g., functional decline, physical frailty), cognitive (e.g., cognitive impairment), and psychological (e.g., depression) domains-or those that investigated interventions aimed at mitigating social frailty were included. A study that examined physical or cognitive frailty without reference to social frailty or those focused on institutionalized elderly populations was excluded from this review.

To ensure the comprehensiveness of the review, all types of empirical studies, systematic reviews, and theoretical discussions relevant to social frailty were included. At the same time, opinion pieces, editorials, and grey literature were excluded. Additionally, the review considered only studies published in English from the inception of each database (PubMed, Embase, and CINAHL) up to 4th November 2024. This focus ensured the synthesis captures global research efforted while maintaining methodological consistency across included studies.

### Information sources and search strategy

A systematic search was conducted across the following electronic databases: PubMed, Embase, and CINAHL, as shown in Table S1.

### Selection of sources of evidence

All identified records were imported into reference management software (EndNote version 21.0) to organize citations and remove duplicates. The selection process followed a rigorous two-stage screening. In the first stage, two independent reviewers (C.H. and W.S.) assessed the titles and abstracts of the retrieved articles to determine their eligibility based on predefined criteria. The predefined criteria were as follows: (1) studies involving community-dwelling older adults (aged 60 years or above); (2) studies addressing the concept, definition, or assessment tools of social frailty; or studies examining its determinants, or associated health outcomes (e.g., disability, mortality, cognitive or physical decline, or psychological distress), or relevant interventions; and (3) articles published in English. Studies including both adults and older adults were allowed for full-text review if they met the other eligibility criteria. Studies not directly related to social frailty among community-dwelling older adults or focused solely on physical or cognitive frailty were excluded. If disagreements arose during this phase, they were resolved through discussion, and if needed, a third reviewer was consulted to reach a consensus (N.B.).

In the second stage, the full texts of potentially relevant studies were retrieved and reviewed in detail to ensure they met all eligibility criteria. This step ensured that only studies addressing social frailty, assessment tools, its determinants, associated outcomes, and relevant interventions were included. Reasons for exclusion at the full-text stage (e.g., focus on institutionalized older adults or absence of social frailty content) were documented to ensure transparency in the selection process.

The selection process was documented using a PRISMA flow diagram, which visually summarized the number of records identified, screened, excluded, and finally included, along with reasons for exclusion during the full-text review stage. This rigorous approach ensures the reliability and reproducibility of the selection process in alignment with PRISMA-ScR guidelines.

### Data charting process

A standardized data charting form was developed and pilot-tested to ensure consistency and accuracy in extracting relevant information from the included studies. The data extracted encompassed several key elements: study characteristics (such as author, publication year, and country), the theoretical frameworks employed, and the measurement tools used to assess social frailty. Additionally, the charting process captured information on the determinants contributing to social frailty and the associated health outcomes, including disability and mortality. Finally, intervention strategies to mitigate social frailty and their reported effectiveness were documented. Details are presented in Table S2.

### Critical appraisal of individual sources of evidence

In accordance with PRISMA-ScR guidelines, we considered whether to conduct a critical appraisal of the included sources of evidence. However, as the objective of this scoping review was to map the existing literature rather than assess the effectiveness or rigor of interventions, we did not perform a formal quality appraisal. This decision is consistent with established scoping review methodology, particularly in reviews incorporating diverse study types (e.g., observational studies, reviews, RCTs), where the goal is breadth rather than depth of evidence assessment [21].

### Synthesis of results

C.H. and W.S. collaboratively synthesized the study results through full-text analysis, adhering to the principles of Data Charting. They organized the narrative synthesis into five key categories: theoretical frameworks, measurement tools, determinants, outcomes, and interventions. This thematic grouping allowed for a structured exploration of social frailty research among community-dwelling older adults. Given the exploratory nature of this scoping review, no formal meta-analysis was conducted. Instead, narrative synthesis provided a comprehensive understanding of the diversity in methodologies and findings, aligned to map the scope of existing evidence and identify knowledge gaps for future research.

Given the limited number of studies directly targeting social frailty interventions identified in the initial search, we included intervention studies that addressed factors related to social frailty, such as social participation, social support, and community engagement. These studies were previously excluded during the screening phase and full-text review due to the criterion of “Not including social frailty as a study variable”. This approach aimed to identify potentially applicable strategies for addressing social frailty.

## Results

**Fig. 1:**
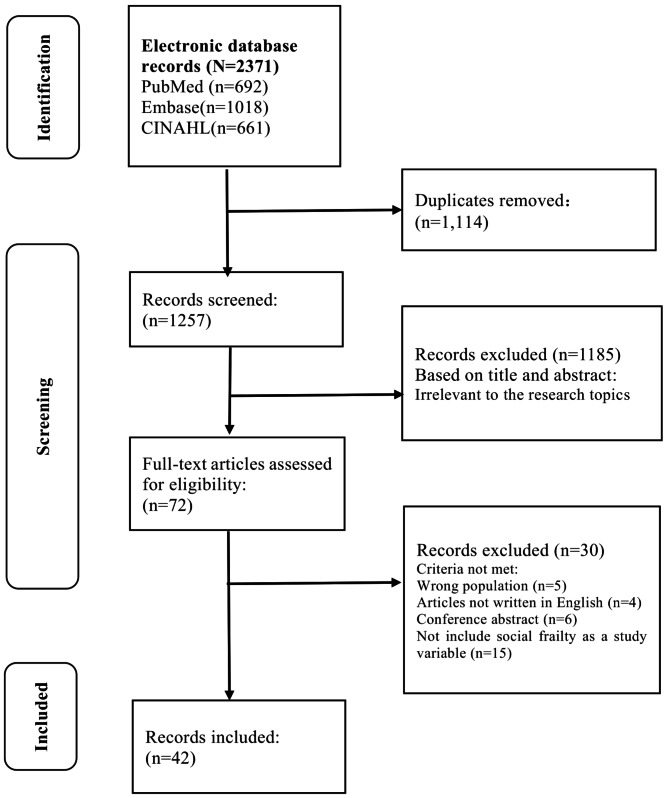
Flowchart of the selection process

Figure 1 illustrates the study selection process. The literature search across electronic databases yielded 2,371 records. 1114 duplicates were removed. In the first screening phase, 1,257 records were evaluated based on their titles and abstracts, resulting in the exclusion of 1185 records. In the second screening phase, the full texts of the remaining 72 documents were further examined, and 30 were excluded. The details of the included and excluded studies in the full-text review are presented in Tables S3 and S4, respectively.

This scoping review identified key themes related to social frailty among community-dwelling older adults, which were organized into five categories. The findings revealed the complexity and multidimensional nature of social frailty, highlighting its interplay with physical, psychological, and cognitive factors. Notably, many of the reported determinants of social frailty also appeared as outcomes, suggesting a potentially reciprocal relationship. Given that most of the included studies employed a cross-sectional design, the reported associations should be interpreted as correlational rather than causal. This conceptual overlap and methodological limitation warrant careful consideration when interpreting the findings. A diverse range of conceptual frameworks and assessment tools were employed across the studies, reflecting ongoing efforts to understand better and measure social frailty. Furthermore, the review underscored the importance of identifying and addressing social frailty to mitigate adverse health outcomes, such as disability and mortality. Various interventions to enhance social participation and support networks have shown promise, although gaps remain in understanding their targeted impact on social frailty and their long-term effectiveness.

### Theoretical frameworks of social frailty

In this review, four articles [3, 16, 22, 23] explicitly defined social frailty, with two studies [16, 22] originating from Canada and two [3, 23] from the Netherlands. Each of these studies proposed a distinct theoretical framework, underscoring the absence of a unified conceptualization in the current literature. The earliest framework, the Deficit Accumulation Model [22], defined social frailty as the proportion of accumulated deficits, reflecting the severity of an individual’s illness and proximity to death based on the volume of these deficits. This model viewed social frailty as a macro-level variable, reflecting general attributes of aging across the whole organism rather than any single functional deficit. It emphasized vulnerability assessment through quantifying accumulated deficits, where higher deficits indicated greater frailty. However, this approach primarily focused on accumulating social deficits and may not fully capture social frailty’s psychological and emotional aspects. The Social Production Function (SPF) Theory [23] explored social frailty by fulfilling three fundamental social needs: emotion, behavioral affirmation, and status. This theory examined how social needs shift with age, physical loss, and subjective well-being, emphasizing the impact of social support and participation in reducing vulnerability. From an emotional and behavioral perspective, it highlighted the role of social interactions in mitigating frailty risk. However, standardized tools based on the SPF theory have not yet been developed, limiting its application and validation across different cultural contexts. The Social Ecology Theory proposed by Andrew et al. [16] provided a socio-ecological approach, focusing on the interaction between individuals and their social environments. Within this framework, SV described the extent to which an individual’s social conditions increase their risk of experiencing various health challenges, including physical, mental, psychological, and functional difficulties [24]. This framework organized an individual’s social environment into four levels and seven aspects, offering insight into preventive and improvement strategies for managing social frailty. Finally, the Social Needs Fulfilment Theory [3], based on the SPF theory, framed social frailty as a continuum influenced by social resources, behaviors, activities, and self-management skills. This theory conceptualized social frailty as a dynamic state dependent on fulfilling social needs, providing a basis for various screening tools used internationally to assess social frailty in older adults [5, 6, 8, 17, 18, 25–27]. The summary of theoretical frameworks is presented in Table 1.

**Table 1:** Theoretical frameworks related to social frailty

Theoretical framework	Operational definition		Related instruments for SF	Reference
Deficit accumulation model	The proportion of accumulated deficits (e.g., symptoms, signs, functional impairments, and laboratory abnormalities) measured by index values can represent an individual’s state at the time of their health assessment.		1. Frailty index 2. Social frailty phenotype 3. Social vulnerability index	Mitnitski, 2001, (Canada) [22]
Social Production Function (SPF) theory	SPF theory specifies three distinctive social needs, which are associated with individual’s well-being. 1. The need for affection 2. Behavioral confirmation 3. Status		None	Steverink, 2006, (Netherlands) [23]
Social Ecology Theory (SET)	SV can be considered at various levels of influence from individual to close family, wider network, and societal context. Seven dimensions of SV based on a SET were proposed as follows:		None	Andrew, 2014, (Canada) [16]
	**SV dimensions**	**SET framework**		
	1. Self-esteem 2. Sense of control	Individual level		
	3. Living situation 4. Relations with others	Interpersonal level		
	5. Engagement 6. Social Support	Community level		
	7. Contextual socio-economic status	Society level		
Social Needs Fulfilment (SNF) theory	SNF can be defined as *“a continuum of being at risk of losing*, *or having lost*, *social and general resources*, *activities*, *or abilities that are important for fulfilling one or more basic social needs during the life span.”* SNF employed a theory of needs, goals, and resources based on SPF theory as a heuristic for ordering and structuring SV in four dimensions as follows: 1. Social needs 2. General resources 3. Social fulfilment 4. Self-management		1. The Makizako Social Frailty Index of 5 items 2. The HALFT scale 3. 4-item social frailty screening index 4. 7-item social frailty index 5. 8-item and 6-item social frailty scale (SFS-8 and SFS-6) 6. Social Frailty Index (SFI) 7. Social Frailty Index (SFI)	Bunt, 2017, (Netherlands) [3]

**Abbreviations**: SF, Social Frailty; SV, social vulnerability; SET, Social Ecology Theory; SPF, Social Production Function theory; SNF, Social Needs Fulfilment theory; SFS, Social Frailty Scale; SFI, Social Frailty Index

### Measurement tools of social frailty

In this review, a total of 13 articles [5, 6, 8, 17, 18, 25–32] proposed the design of measurement tools for social frailty, with contributions from Australia [28–30] (3 articles), Japan [6, 8], Singapore [17, 26], and China [5, 18] (2 articles each), and Canada [31], Spain [32], Korea [25], and India [27] (1 article each). The tools developed in these studies are grounded in distinct theoretical frameworks, allowing for categorization into two main types: those based on the Deficit Accumulation Model, which conceptualizes frailty as a cumulative process of deficits, and those based on the Social Needs Fulfilment Theory, which emphasizes the role of social resources and engagement in mitigating frailty.

From Table 2, four measurement tools have been created based on the Deficit Accumulation Model. The SVS-22 [28] focuses on assessing the financial vulnerability of older adults. Developed from this, the SVS-15 [29, 30] offers a more concise and user-friendly assessment process, primarily focusing on the vulnerability of older adults to exploitation, providing a more precise assessment of social frailty, particularly in cognitive function and psychosocial aspects. The SVI [31] was comprehensive, considering multiple dimensions, focusing on assessing community and group social frailty, suitable for secondary analysis of large-scale public health data. The SVI showed good predictive value for the mortality of older adults [31]. The SVI has been culturally validated in Dutch [33] and Tanzanian [34] language and cultural settings. The SFP [32] was primarily concerned with the insufficiency of social function in older adults, emphasizing individual-level characteristics and states of social frailty. The SFP had concise items that help predict health risks in older adults, although some items lack clear definitions, adding some difficulty to the assessment. The scores of the above measurement tools (excluding the SFP) range from 0 to 1, with scores closer to 1 indicating a higher degree of social frailty.

**Table 2:** Measurement tools for social frailty (Based on deficit accumulation Model)

Measurement tools	Dimensions/phenotypes (items)	Scoring structure	Interpretation	Reference
**Theoretical framework**: Deficit Accumulation Model				
22-item Social Vulnerability Scale (SVS-22)	4 dimensions (22 items) 1. Financial decisions 2. Trusting strangers 3. Following instructions 4. Susceptibility to scams	5-point Likert scale (range 0–88) 4 = always 3 = often 2 = sometimes 1 = rarely 0 = never	Higher scores indicate greater social vulnerability	Pinsker, 2006 (Australia) [28]
15-item Social Vulnerability Scale (SVS-15)	2 dimensions (15 items) 1. Gullibility (8 items) 2. Credulity (7 items)	5-point Likert scale (range 0–60)	Higher scores indicate greater social vulnerability	Pinsker, 2011 (Australia) [29, 30]
Social Vulnerability Index (SVI)	5 dimensions (40 items) 1. Social engagement 1.1. Communication (2 items) 1.2. Living situation (2 items) 2. Social support (12 items) 3. Daily living items/ Daily social activity 3.1 Socially oriented activities of daily living (2 items) 3.2 Leisure activities (6 items) 4. Sense of mastery/ control over one’s life circumstances 4.1 psychological well-being (4 items) 4.2 A feeling of life (9 items) 5. Socio-economic status (3 items)	SVI was calculated as a proportion of the total number of deficit items by dividing the sum of deficit scores by the number of deficits considered. (range 0–1) Scoring interval 0–1 on each deficit For example: - binary rating on social deficits 1 = present 0 = absent - three categories of social deficits 1 = often 0.5 = sometimes 0 = never - five categories of social deficits would be scored 0, 0.25, 0.5, 0.75, and 1	Higher scores indicate greater social vulnerability	Andrew, 2008 (Canada) [31]
Social frailty phenotype (SFP)	6 phenotypes 1. Living alone 2. Lack of a person to help with activities of daily living 3. Infrequent contact with family 4. Infrequent contact with friends or neighbors 5. Absence of a confidant 6. Lack of support for daily living during the past 3 months	Binary rating on social frailty phenotypes 1 = present 0 = absent	≥ 2 phenotypes indicate social frailty status	Garre-Olmo, 2013 (Spain) [32]

**Abbreviations**: SVS, Social Vulnerability Scale. SVI, Social Vulnerability Index. SFP, Social Frailty Phenotype

Measurement tools constructed based on the Social Needs Fulfilment Theory focus more on understanding how older adults meet social needs through social participation and resources, as shown in Table 3. The Social Frailty Index (5 items) by Makizako et al. [8] evaluated basic social activities and emotions in elderly Japanese, effectively predicting disability and mortality, making it suitable for large prospective studies. A simplified 4-item version further reduces cognitive load, enabling quick screening in community settings [6]. The 7-item index assesses multiple social frailty dimensions, aiding early detection of depressive symptoms [17]. The HALFT scale (5 items) [5] emphasized the impact of economic and social activity, and it has been adapted for Brazilian used [35]. The Social Frailty Scale (5 components) [25] linked social frailty to hearing loss, adding a sensory health perspective. The 8-item scale [26] use three factors (social resources, activities, and needs), predicting outcomes like mood and nutrition independently of physical frailty. The 6-item scale [18] included family harmony, balancing thoroughness with ease of response, and effectively forecasting depressive symptoms. These measurement tools have clear cutoff scores for levels of social frailty, facilitating the identification and stratification of social frailty severity. Lastly, the Social Frailty Index was a newly developed scale that measures various aspects of older adults’ family resources, social capital, social behavior, and social activities, providing a comprehensive assessment of social frailty. A higher score indicated a higher level of social frailty.

**Table 3:** Measurement tools for social frailty (Based on social needs fulfilment Theory)

Measurement tools	Dimensions/phenotypes (items)	Scoring structure	Interpretation	Reference
**Theoretical framework**: Social Needs Fulfilment (SNF) theory				
The Makizako Social Frailty Index of 5 items	3 dimensions (5 items) 1. Daily social activity (1 item) 2. Social role (1 item) 3. Social relationships (3 items)	The score was the sum of 5 items (range 0–5) Binary rating on each item (yes/no)	0 indicates non-social frailty 1 indicates pre-social frailty ≥ 2 indicates social frailty	Makizako, 2015 (Japan) [8]
7-item social frailty index (SFI)	2 dimensions (7 items) 1. General/social resources (4 items) 2. Social behaviors and activities (3 items)	The score is the sum of 7 items (range 0–7) Binary rating on each item 1 = yes/present 0 = no/absent	0 indicates no social frailty 1 indicates low social frailty ≥ 2 indicates social frailty	Teo, 2017 (Singapore) [17]
HALFT scale	5 items 1. **H**elp others (Social role) (1 item) 2. Participation (Social activity) (1 item) 3. **L**oneliness (1 item) 4. **F**inancial (Socio-economic status) (1 item) 5. **T**alk (Social relationships) (1 item)	The score is the sum of 5 items (range 0–5) Binary rating on each item (yes/no)	0 indicates non-social frailty 1–2 indicate pre-social frailty ≥ 3 indicates social frailty	Ma, 2018 (China) [5]
4-item social frailty screening index	4 items 1. General resources (1 item) 2. Social resources (1 item) 3. Social behavior (1 item) 4. Fulfillment of basic social needs (1 item)	The score is the sum of 4 items. (range 0–4) Items 1, 3, and 4 are 4-point Likert scale 4 = very satisfied 3 = satisfied 2 = dissatisfied 1 = very dissatisfied (“very dissatisfied” assign a score of 1) Item 2 is answered by binary rating. 1 = yes 0 = no	0 indicates socially robust 1 indicates pre-social frailty ≥ 2 indicates social frailty	Yamada, 2018 (Japan) [6]
Social frailty scale(SFS)	5 items from (Makizako, 2015) 1. Neighborhood meeting attendance 2. Talking to friend(s) 3. Someone gives you love and affection 4. Living alone 5. Meeting someone every day	The score is the sum of 4 items. (range 0–5) The answer of “yes” to the item 4 and “no” to the items 1,2,3,5 were considered as 1 score.	0 indicates non-social frailty 1–2 indicate pre-social frailty ≥ 3 indicates social frailty	Yoo, 2019 (Korea) [25]
8-item social frailty scale (SFS-8)	3 dimensions (8 items) 1. Social resources (3 items) 2. Social activities and financial resources (3 items) 3. Social need fulfillment (2 items)	The score is the sum of 8 items. (range 0–8) Binary rating on each item (yes/no)	0–1 indicates non-social frailty 2–3 indicate pre-social frailty ≥ 4 indicates social frailty	Pek, 2020 (Singapore) [26]
6-item social frailty scale (SFS-6)	4 dimensions (6 items) 1. Social support (2 items) 2. Social isolation (2 items) 3. Social activity (1 item) 4. Economic status (1 item)	The score is the sum of 6 items. (range 0–6) Binary rating on each item (yes/no) 1- point for a negative response	0–1 indicates non-social frailty 2–3 indicate pre-social frailty ≥ 4 indicates social frailty	Chen, 2021 (China) [18]
Social Frailty Index (SFI)	5 dimensions(17items) 1. Decision making participation (5 items) 2. Social network(friends)(3items) 3. Social activity(4items) 4. Social network(others)(2items) 5. Social activity at community level (3 items)	The indicator was coded in binary (1 = Yes and 0 = No) or quintile form (Daily/several times a week = 1; Once a week/several times a month = 0.75; At least once a month = 0.50; Rarely in a year = 0.25; never/not relevant = 0 ) then standardizing their responses on a scale of 0 to 100. (sum of all indicators and further harmonized into range 0–100)	A higher score indicates a high level of social frailty.	Irshad, 2024, (India) [27]

**Abbreviations**: SNF, Social Needs Fulfilment theory; SFI, Social Frailty Index; SFS, Social Frailty Scale

For secondary data analysis, we advise utilizing longer scales with more items (such as SVI and SFI), while for initial data collection, shorter scales with fewer items (such as SFP, MSFI, SFI, and HALFT) are recommended. All these scales rely on participants’ self-reports, which provide flexibility across various times and settings, making it easy to complete assessments. However, they can be influenced by potential biases from personal perceptions and memory recall, which may affect the accuracy of the results.

### Determinants of social frailty

In this review, 12 articles [27, 36–46] examine the determinant factors of social frailty, including contributions from Japan [36–41] (6 articles), Northland [42–44] (3 articles), and Australia [45], India [27], and Canada [46] (1 article each). The identified determinants are categorized into five main domains: physical, cognitive, psychological, lifestyle, and health-related factors. The summary of determinants of social frailty is presented in Table S5.

### Physical function and physical frailty

Two studies indicated a significant association between physical frailty and social frailty [36, 37]. Regarding this association, Inoue et al. [36] described that older adults with physical frailty, such as declines in muscle strength and bone density, had an increased risk of social frailty. However, their findings suggested a reciprocal relationship, indicating that social frailty may also contribute to physical decline. Their cross-sectional analysis implied that social frailty could accelerate physical deterioration by reducing physical activity and increasing isolation, ultimately exacerbating conditions such as osteosarcopenia. In a two-year prospective cohort study, older adults with physical frailty significant increased risk of social frailty with the potential confounder adjustments, including age, sex, depression, multimorbidity, daily and cognitive function [37]. Slow gait speed and weakness were also identified as independent risk factors for development of social frailty, as social frailty assessed by social activity and contact with neighbors. Their findings indicated that physical frailty was a cause of social frailty. This longitudinal data supported the hypothesis that physical frailty was an early catalyst for social decline, primarily through mobility limitations and reduced social engagement. These results indicated that the physical health status of older adults has a crucial impact on the development of social frailty.

### Cognitive functions

Two articles reported the impact of cognitive function on social frailty [27, 46]. Data from the Canadian Study of Health and Aging, a cross-sectional study including 3,776 study participants, indicated a positive association between cognitive impairment and social frailty, where higher cognitive decline associated with increased social frailty [46]. However, as a cross-sectional study, it cannot establish causality, and reliance on self-reported data may introduce recall bias. Irshad analyzed data from Wave 1 of the Longitudinal Aging Study in India (LASI), a nationwide prospective study spanning 34 states and union territories (excluding Sikkim), and found that among 23,361 older adults, poor cognitive health was linked to an increased risk of social frailty, as cognitive impairments can hinder social engagement and weaken social resilience [27]. While LASI’s prospective design is a strength, the study lacks follow-up data, limiting causal inference. Additionally, the newly developed Social Frailty Index (SFI) requires further validation for broader application. These studies suggested a link between cognitive function and social frailty, but causality remains unclear.

### Psychological health

Three studies examined the association between depression and social frailty, identifying depression as an independent risk factor for social frailty [27, 38, 39]. In a cross-sectional study in rural northern Japan, Kume et al. [39] observed that social frailty independently correlates with depressive symptoms in this population. Additionally, decreasing social participation among older adults has been shown to heighten depressive symptoms, which further raises social frailty risk [38]. Irshad et al. demonstrated that depressive symptoms exacerbate social frailty by limiting social interactions and support [27]. In summary, these findings underscore the importance of monitoring both cognitive and mental health in older adults as a strategy for mitigating social frailty risks.

### Lifestyle factors

Regarding lifestyle factors, two studies focused on physical activity [42, 43], two examined the impact of sleep duration [40, 42], and one explored social behavior in relation to social frailty [45]. For physical activity, van Oostrom et al. [42] highlighted that engaging in at least 30 min of moderate to vigorous physical activity daily, five days a week, significantly lowers the risk of frailty across physical, psychological, cognitive, and social domains. Similarly, Ye et al. [43] report that infrequent physical activity (once a week or less) correlates with a higher risk of social frailty.

Sleep duration also plays a critical role in the well-being of older adults. Studies [40, 42] indicated that both insufficient (≤ 5 h) and excessive (≥ 9 h) sleep durations are linked to elevated frailty risks across these exact domains. Specifically, Nakakubo et al. [40] found that excessive daytime sleepiness correlated with a higher likelihood of social frailty, likely due to its negative impact on physical function, cognitive decline, and mental well-being. Specifically, prolonged sleep duration has been associated with reduced mobility and fatigue, limiting opportunities for social participation [40]. Cognitive impairments resulting from poor sleep patterns could further hinder social engagement, communication, and relationship maintenance [42]. Although disrupted sleep has been linked to depressive symptoms and anxiety, both of which are associated with social withdrawal and isolation, it may not necessarily serve as a contributing factor but rather represent a symptom of underlying psychological conditions [40]. These findings suggest a link between sleep duration and social frailty; however, cross-sectional designs and self-reported sleep measures limit causal inference, requiring further validation through longitudinal studies.

Additionally, Henry et al. [45] indicated that inappropriate social behavior may lead to negative reactions from others toward the older adult, thereby weakening their social network and support, thus increasing social frailty risk. Overall, these findings suggest that regular physical activity, balanced sleep, and positive social behavior are vital lifestyle factors, with deficiencies in these areas potentially elevating the risk of social frailty in older adults.

### Other health indicators

In terms of other health indicators, two articles emphasized the association of pain with social frailty [30, 37], while another two examined the influence of multimorbidity, with one also addressing the effect of medication risk [43, 44]. Research indicated that moderate to severe pain, especially chronic pain with significant symptoms persisting for at least six months, negatively impacts older adults’ daily social interactions, physical functioning, and mental health, gradually reducing their engagement with others and ultimately increasing the risk of social frailty [41, 46]. Ye et al. [43] found that health complications arising from multimorbidity often restrict social involvement, thus markedly increasing the risk of social frailty among older adults. Similarly, Gobbens et al. also identified an association between multimorbidity and social frailty, although this link was comparatively weaker [44]. Additionally, multimorbidity often leads to increased medication use, which in turn raises medication-related risks. The study by Ye et al. suggested that older adults with polypharmacy were significantly more likely to experience frailty across physical, psychological, and social domains, with improper medication use particularly associated with an elevated risk of social frailty [43]. The study assessed medication risk using the Medication Risk Questionnaire (MRQ-10), which evaluates inappropriate medication use, polypharmacy, and potential adverse drug reactions. However, it did not explicitly specify which types of medications or specific side effects contribute to social frailty. Pain, multimorbidity, and medication risks collectively amplified social frailty in older adults by limiting social interaction and increasing vulnerability across multiple domains.

### Consequence of social frailty

In this review, 22 articles [5, 6, 8, 17, 18, 26, 31, 32, 34, 45, 47–58] examine the consequence of social frailty, including contributions from Japan [6, 8, 47, 48, 50–53, 58] (9 articles), China [5, 18, 49, 54] (4 articles), Singapore [17, 26], Canada [31, 55], Korea [56, 57] (2 articles each), and Spain [32], Australia [45], and Tanzania [34] (1 article each). Social frailty is closely linked to various adverse health outcomes, notably higher risks of disability, mortality, physical functional decline, cognitive decline, and depression. The details of social frailty consequences are summarized in Table S6.

### Morbidity and mortality

In this scoping review, a total of 12 articles examined the impact of social frailty on morbidity and mortality [5, 6, 8, 17, 31, 32, 34, 47–49, 56]. Many studies indicated that older adults with social frailty are more likely to experience disability and severe dependency compared to those without social frailty [8, 17, 47, 48]. Furthermore, social frailty increased the need for assistance in Activities of Daily Living (ADLs) and ADL-related disabilities [56]. Over follow-up periods of two and six years, socially frail individuals were at greater risk of developing functional and severe disabilities, leading to increased dependency on daily activities and a diminished quality of life [6, 8, 56].

Research consistently showed that social frailty significantly increased mortality risk among older adults [5, 6, 31, 32, 34, 48, 49]. A longitudinal study in Japan [6] and two cross-sectional studies [5, 49] found that socially frail older adults had a higher risk of death within six years [6]. Yamada et al. [6] reported a 66.1% incidence of death or disability among individuals with both social and physical frailty (HR = 2.29), while Sun et al. [49] found a 6-year mortality rate of 13.7% among socially frail individuals (OR = 2.22). Ma et al. [5] further showed that social frailty predicted 8-year mortality, with adjusted hazard ratios ranging from 2.5 to 4.3. These studies also indicated that the coexistence of social and physical frailty limits access to essential resources such as nutrition and healthcare, reduces social support, and diminishes self-care abilities, thereby heightening risk [6, 49]. Thus, social frailty raises the risk of disability and mortality, contributing to greater dependency and reduced survival rates.

### Physical function and physical frailty

Three articles demonstrated the impact of social frailty on physical functioning [50, 51, 58]. A four-year longitudinal study by Makizako et al. [58] revealed that individuals with social frailty faced a higher risk of progressing to physical frailty. Additional studies indicated that older adults with social frailty were more susceptible to hand grip strength [50]. Similarly, Huang et al. [51]identified a strong association between social frailty and decline in intrinsic capacity, including reduced grip strength and poorer nutritional status, contributing to more significant physical frailty and dependency on daily activities. The included studies revealed that social frailty significantly impaired physical functioning, raising the risk of muscle weakness, physical decline, and dependence among community-dwelling older adults.

### Cognitive functions

A total of three articles highlighted the impact of social frailty on cognitive function [51, 54, 55]. The evidence regarding the association between cognitive decline and social frailty demonstrated that individuals with higher levels of social frailty were at a higher risk for cognitive decline [55]. The findings from the Honolulu-Asia Aging Study [55] found that higher levels of social frailty predicted cognitive decline over three and six years. Similarly, Huang et al. [51] observed a connection between social frailty and diminished cognitive and psychological function, particularly in men, indicating that socially frail men experienced a more significant decline. Additionally, Zhang et al. [54] reported that older adults with social frailty were at higher risk of developing Motoric Cognitive Risk (MCR) syndrome, which affected both subjective cognitive complaints (without dementia) and slowed gait speed. These findings highlighted the importance of addressing the impact of social frailty on cognitive function.

### Psychological health

A total of eight articles mentioned the impact of social frailty on psychological health [18, 26, 45, 51–53, 56, 57]. Studies consistently showed that social frailty significantly contributed to the development of depression [18, 26, 51, 52, 56]. Chen et al. [18] confirmed that social frailty was independently associated with worsening depressive symptoms in Chinese older adults with depression; those without depression were also associated with an increased risk of developing symptoms after three years. Additionally, studies indicated that older adults with social frailty have a higher risk of depressive symptoms compared to their non-frail peers [26]. Furthermore, social frailty is linked to greater demoralization, lower psychological resilience, and reduced life satisfaction [45, 57]. Noguchi et al. [53] also found that social frailty correlates with poorer sleep quality. Overall, social frailty is strongly associated with adverse psychological outcomes, especially depression and reduced mental well-being in older adults.

### Intervention strategies

This review identified only one randomized controlled trial (RCT) examining digital health interventions, which showed no significant improvement in social frailty [59]. Nonetheless, the interconnected nature of physical, psychological, and social frailty suggests that certain general frailty interventions may also be relevant to social frailty. Consequently, this review summarized closely related research on broader frailty interventions to identify potentially suitable strategies for social frailty and interventions targeting specific aspects of social frailty, including physical exercise programs, social network enhancement, and digital health interventions.

### Physical exercise interventions

The review summarized three relevant studies on physical exercise interventions. One study examined that a Real-Life exercise intervention for frail older adults, which included strength exercises, cardiorespiratory exercises, neuromotor training, and stretching, conducted three times a week for 60 min over 24 weeks, led to significant improvements in functional parameters, dietary habits, emotional well-being, and social network outcomes [60]. This study was conducted in a real-life community setting, enhancing its generalizability and feasibility. However, the lack of a strict control group and limited long-term follow-up weaken its ability to establish causal relationships. Similarly, a study on a multicomponent exercise program (MEP) for frail community-dwelling older adults involved proprioception, aerobic, strength, and stretching exercises, each lasting 65 min, performed five days a week for 24 weeks. This intervention effectively reversed frailty and improved cognition, emotional well-being, and social networking [61]. This RCT supported the effectiveness of multicomponent exercises in improving social networking, but practical challenges in real-world implementation and long-term sustainability need further research. In addition, a yoga-based lifestyle intervention aimed at healthy aging demonstrated that a 26-week yoga program significantly improved aging biomarkers and positively influenced social well-being, including better social relationships and reduced loneliness [62]. This study suggested potential benefits for social frailty. However, self-reported adherence and cultural specificity limit its generalizability. These findings emphasized that comprehensive exercise programs could enhance older adults’ social networks and relationships, potentially helping to alleviate social frailty.

### Social networking enhancement

The review summarized three studies focused on enhancing social networks [63–65]. Harada et al. showed that robust community networks, built on social relationships, support, and interactions, enhance elderly health behaviors like daily activities, living arrangements, and self-rated health [63]. This study demonstrated the long-term benefits of social engagement; however, its non-randomized design limited the ability to establish causality. A study in Thailand revealed that including family members in monthly meetings and activities for older adults over a 12-month period led to a significant improvement in overall quality of life, social engagement, and social support among older adults, along with a reduction in depression rates [64]. This suggested that family network-based health promotion activities could effectively improve older adults’ mental health and quality of life. And a larger-scale RCT was needed to confirm findings and test long-term sustainability. Additionally, a three-year intervention involving monthly community activities increased social participation among community-dwelling older adults, improved their social relationships, strengthened community networks, and fostered closer connections with neighbors, all of which positively impacted the mental health of older adults [65]. However, the study’s lack of randomization and reliance on self-reported data limit its causal validity. In conclusion, building strong social networks and encouraging regular social participation can significantly improve the health and well-being of older adults.

### Digital health interventions

Only one RCT by Pollak et al. [59] was exploring the effects of a robotic pet on social frailty using the Questionnaire to Define Social Frailty Status(also named the Makizako Social Frailty Index of 5 items, which was proposed by Makizako [8]) in community-dwelling older adults recently discharged from the hospital. However, the results indicated no significant decrease in the incidence of social frailty, likely due to the short intervention duration (1 month) and lack of specific guidance on pet interaction frequency.

There were three studies on digital health interventions using electronic devices to promote health among older adults in the community [66–68]. One study in Japan examined the mobile health (mHealth) application “Online Kayoinoba”, which enhanced physical activity levels, improved cognitive function, and increased social interaction among older adults through a 6-week intervention course followed by a 7-week observation period [66]. The frailty phenotype scores and step counts improved after intervention. This app included a communication feature, along with viewpoints and step-ranking functions, highlighting its social networking capabilities. These features may have enhanced users’ awareness of their social connections and helped strengthen those connections. Additionally, the study employed smartwatches and mobile applications to remind older adults who did not meet the daily recommended physical activity levels, thereby promoting their self-management of exercise and subsequently improving both physical and cognitive health [67]. In another study involving community-dwelling older adults, a multidisciplinary telehealth approach, with remote care from multiple specialists, demonstrated reductions in caregiver burden and improvements in mood, behavior, activities of daily living/instrumental activities of daily living, and nutritional status over time [68].

By enhancing older adults’ physical health, exercise self-management, psychological well-being, and quality of life, the aforementioned interventions could support the development of general resources and self-management capabilities.

## Discussion

We conducted a scoping review on social frailty among community-dwelling older adults, highlighting its complex and multidimensional nature as captured by various theoretical frameworks. However, existing measurement tools vary in focus and application, addressing different aspects of social frailty yet lacking region-specific cross-cultural validation and standardized, unified instruments. The studies in this review suggested that factors such as physical and cognitive function, psychological health, and significant life events elevated the risk of social frailty in older adults. Moreover, social frailty was confirmed to be linked to adverse outcomes like disability, cognitive decline, and increased mortality rates. Although interventions aimed at social support, community participation, and digital health solutions may be beneficial, further research using standardized measurements is needed to assess their effects on social frailty. These findings underscore the need for a standardized approach to evaluating and addressing social frailty to promote healthy aging and improve the quality of life for older adults in community settings.

### Conceptual framework of social frailty

Among the four included articles that explicitly defined social frailty, three were grounded in empirical research [16, 22, 23], while one [3] offered a theoretical synthesis derived from a scoping review. This distinction highlights the evolving nature of social frailty as a construct shaped by both empirical evidence and conceptual development. By analyzing the theoretical framework of social frailty, specific guidance can be proposed across different dimensions. Social frailty highlights the importance of social relationships, emotional support, and self-management, offering a foundation for designing interventions to strengthen community networks and enhance individual social participation [63]. Such an integrative perspective improves the scientific rigor and practicality of policy-making and supports the development of more effective strategies to prevent and manage social frailty.

However, existing conceptual models of social frailty vary in scope and applicability, posing challenges for integration. The Deficit Accumulation Model quantifies frailty through accumulated deficits but overlooks psychological and emotional aspects. The SPF theory focuses on social needs but underestimates other key dimensions of social resources, resulting in a limited evaluative scope and the absence of a standardized assessment scale for measuring social frailty. The SET highlights structural influences but remains difficult to operationalize for targeted interventions. Most available measurements for social frailty were based on the SNF theory, which conceptualizes social frailty as a dynamic continuum. However, these measurements primarily served as cross-sectional screening tools to identify individuals at risk, rather than capturing the dynamic changes in frailty over time. Despite their contributions, these frameworks remained theoretical with limited empirical validation and implementation and lacked connections to intervention strategies.

Identifying key social resources and support networks provides a crucial basis for shaping policies that address these vulnerabilities, particularly in global aging. Further synthesis and application of existing theoretical frameworks can empower public health initiatives to address the complex needs of older adults better, promote healthy aging, and mitigate the growing public health burden of social frailty.

### Measurement tools

Overall, social frailty assessment scales are essential tools for identifying the social frailty of older adults. Future research should further standardize these scales to ensure their cross-cultural applicability, validating their universality in broader populations, including older adults from diverse cultural backgrounds and socioeconomic statuses. Additionally, integrating theoretical frameworks into constructing assessment tools can enhance their robustness and relevance, allowing for a deeper understanding of the underlying dimensions of social frailty.

However, existing measurement tools have notable limitations. Most scales primarily assess the fulfillment of social needs, roles, and resources [6, 8, 17], often overlooking the psychological well-being of older adults. While some tools evaluate psychological conditions [5, 18], they tend to emphasize negative emotions while neglecting key positive self-beliefs such as self-esteem and self-efficacy. Moreover, the assessment of social frailty in older adults with psychological issues remains insufficient, as no standardized criteria have been established for this population, underscoring a critical research gap. Expanding these domains would improve the sensitivity and comprehensiveness of these tools, enabling a more holistic evaluation of social frailty.

As social structures and the needs of older adults evolve, the content of these scales may require updates to reflect emerging social issues and risk factors more accurately. Moreover, future research should focus on integrating social frailty assessment scales into intervention frameworks, using scale results to design and evaluate preventive interventions that could slow down or even reverse the process of social frailty. Advances in technology offer promising avenues to integrate social frailty assessments with electronic health records and remote monitoring, enabling efficient data collection, continuous tracking, and real-time intervention adjustments.

### Determinants and adverse outcomes of social frailty

A clear interconnection emerges in summarizing the factors contributing to social frailty and its negative outcomes. Factors including physical decline, cognitive impairment, depressive mood, lifestyle choices, and disease-related conditions can accelerate social frailty. As social frailty worsens, the risk of adverse outcomes, such as disability, further physical and cognitive decline, and depressive symptoms, also rises, creating a vicious cycle. This cycle accelerates frailty and complicates recovery, highlighting the need for comprehensive intervention strategies.

### Physical function and physical frailty

Physical function and physical frailty are both influential factors in social frailty and its progression. With aging, declining physical capacity can increase social isolation and vulnerability [45, 69], while social frailty may, in turn, worsen physical decline by reducing motivation for activity and diminishing support networks. Limited social engagement can isolate older adults, accelerating physical deterioration and raising risks of disability and mortality. This bidirectional relationship underscores the importance of addressing physical frailty in social frailty interventions, potentially breaking this negative feedback loop.

### Cognitive functions

Cognitive function is central to social frailty, with cognitive impairment affecting 30–50% of community-dwelling adults over 85 [70]. Conditions such as mild cognitive impairment and dementia disrupt thinking, memory, and attention, leading to more than typical age-related cognitive changes. As cognitive decline progresses, individuals’ social structures often weaken, limiting social interactions and heightening frailty. Conversely, the social isolation linked to social frailty can further accelerate cognitive decline, potentially contributing to dementia [55]. Therefore, incorporating cognitive health into social frailty interventions may foster social engagement and bolster cognitive resilience.

### Psychological health

Depression is both a cause and a consequence of social frailty [18, 39]. Individuals lacking social support or experiencing loneliness are more prone to depressive symptoms, while a depressive state often leads them to withdraw from social activities, further intensifying social frailty. During the COVID-19 pandemic, depressive symptoms among older adults worsened, confirming the significant impact of social participation on mental health [38]. This cyclical relationship highlights the essential role of mental health support in interventions for social frailty, as it can enhance social engagement and reduce the risk of adverse psychological outcomes.

### Adverse outcomes - morbidity and mortality

Current evidence indicates that social frailty significantly impacts morbidity and mortality rates among older adults, with mortality being the most critical adverse health outcome. Older adults with social frailty have a 2–4 times higher risk of mortality compared to their non-frail peers [49]. Additionally, socially frail individuals face a 1.3 to 6.3 times higher risk of functional and severe disability [17]. These findings underscore the significant impact of social frailty on older adults’ health, emphasizing the urgent need to enhance social support and engagement for this population. Social frailty not only increases the risk of illness and mortality but also diminishes self-care abilities and quality of life. Often accompanied by insufficient social support, heightened isolation, and reduced health-promoting behaviors, social frailty creates a vicious cycle that exacerbates health challenges for older adults.

### Other aspects of determinants

Both excessive and insufficient sleep are associated with a higher risk of social frailty [42]. Short sleep durations may lead to physical fatigue, decreased immunity, and cognitive function decline, while long sleep durations may be related to chronic health problems. Sleep quality affects mood, cognitive function, and physical strength, all of which are crucial for social participation. By addressing sleep habits in interventions for social frailty, it may be possible to improve emotional and cognitive resilience, thereby reducing the overall health impact of social frailty.

Chronic pain [41, 46] and multimorbidity [43] restrict older adults’ social engagement, further weakening social support networks. These factors are closely associated with severe adverse outcomes, such as disability and mortality. Managing chronic diseases could improve older adults’ physical health and potentially increase social participation, alleviate emotional and cognitive decline, and thus mitigate social frailty’s adverse effects. Effective care strategies should include optimizing multimorbidity management to minimize medication side effects, promoting balanced nutrition and physical activity to maintain physical functions, and fostering social support networks. Interdisciplinary collaboration is essential for developing personalized, holistic care plans to lower the incidence of social frailty and enhance older adults’ quality of life through sustained social participation and support [71, 72].

### Intervention strategies

Research showed that regular exercise lowered the risk of social frailty in older adults [42], primarily by fulfilling social role expectations and enhancing self-management. These benefits are achieved by improving physical health, reducing falls, and enhancing mental well-being through reduced symptoms of depression and anxiety. Group or community-based exercise programs are particularly effective, fostering social connections that help combat isolation and build resilience [65]. Family support and community activities strengthen social networks, fostering social resilience and reducing frailty risks by supporting interpersonal and community engagement.

Beyond physical activity and social network enhancement, digital health technologies present new opportunities for addressing social frailty. Digital health apps [66], wearable devices [67], and telemedicine [68] offer innovative ways to support both physical and social health. However, current research on these technologies’ effectiveness in mitigating social frailty is limited, highlighting a need for further investigation.

Future research should focus on developing and validating targeted, multidimensional interventions that integrate exercise and social engagement while utilizing community resources to counteract social isolation. Digital health interventions like telemedicine and mobile health apps offer personalized, adaptive support, enabling real-time monitoring and intervention, which is particularly valuable for those who are homebound or in remote areas.

Yet, as technology use grows among older adults, so do risks, including fraud and privacy concerns. Thus, safety education must accompany the promotion of digital health tools to enhance older adults’ awareness and empower them to benefit from digital advances safely.

### Limitations

This scoping review provides valuable insights into social frailty among community-dwelling older adults but faces some limitations. First, the review included only studies published in English, potentially overlooking relevant findings available in other languages. As a result, the applicability of the findings to non-English-speaking populations may be limited, as factors influencing social frailty, as well as effective interventions, could vary across cultural contexts. Second, although this review focused specifically on studies using the term “social frailty” and “social vulnerability”, we excluded related concepts such as loneliness, social support, and social isolation from the keyword search to maintain conceptual precision. This decision may have limited the comprehensiveness of the review by omitting studies that, while not using the term explicitly, addressed overlapping constructs and potentially relevant evidence. Finally, due to the lack of direct intervention studies on mitigating social frailty, the summarized results for the intervention strategies should be interpreted with caution.

## Conclusions

This scoping review highlights the need for standardized, culturally adaptable tools to assess social frailty among community-dwelling older adults. Current tools are based on two primary frameworks: the Deficit Accumulation Model, effective for secondary data and screening, and the Social Needs Fulfillment Theory, which utilizes brief-item tools but requires further context-specific validation. Social frailty is linked to increased morbidity and mortality, yet specific interventions remain undeveloped. Future research should prioritize creating and validating targeted interventions, such as structured exercise, social network support, and digital health solutions, to address the multidimensional aspects of social frailty and break its negative feedback loop.

## Electronic supplementary material

Below is the link to the electronic supplementary material.

Supplementary Material 1

Supplementary Material 2

## Data Availability

No datasets were generated or analysed during the current study.

## Abbreviations

SF: Social Frailty
SV: Social Vulnerability
SET: Social Ecology Theory
SPF: Social Production Function theory
SNF: Social Needs Fulfilment theory
SFS: Social Frailty Scale; SFI, Social Frailty Index
SVS: Social Vulnerability Scale
SVI: Social Vulnerability Index
SFP: Social Frailty Phenotype
SFI: Social Frailty Index
SFS: Social Frailty Scale
