# Supplementary material for: Social frailty in community-dwelling older adults: a scoping review
Source: BMC Geriatr. 2025 May 13;25:329. doi: 10.1186/s12877-025-05971-0 (PMC12070721; doi:10.1186/s12877-025-05971-0)
Supplement: Supplementary file 1 — Supplementary Material 1 [file 12877_2025_5971_MOESM1_ESM.docx]

**Table S1. Search strategy for the scoping review**

| **Database** | **Search Terms** | **Search Strategy** | **Initial Search** | **Last Search** |
| --- | --- | --- | --- | --- |
| **PubMed** | **#1:(((social frailty[Title/Abstract]) OR (social frail*[Title/Abstract])) OR (social vulnerability[Title/Abstract])) OR (social vulnerabilit*[Title/Abstract])**  **#2:(((((("Aged"[Mesh]) OR (elder[Title/Abstract])) OR (elder*[Title/Abstract])) OR (senior*[Title/Abstract])) OR (geriatric*[Title/Abstract])) OR (older adult*[Title/Abstract])) OR (older people[Title/Abstract])** | **#1 AND #2** | (*n* = 542; Mar 15, 2024) | (*n* = 686; Nov 4, 2024) |
| **Embase** | **#1:'social frailty'/exp OR 'social frail*':ab,kw,ti OR 'social vulnerability':ab,kw,ti OR 'social vulnerabilit*':ab,kw,ti**  **#2:****'aged'/exp OR 'aged patient':ab,kw,ti OR 'aged people':ab,kw,ti OR 'aged person':ab,kw,ti OR 'aged subject':ab,kw,ti OR 'elderly':ab,kw,ti OR 'elderly patient':ab,kw,ti OR 'elderly people':ab,kw,ti OR 'elderly person':ab,kw,ti OR 'elderly subject':ab,kw,ti OR 'senior citizen':ab,kw,ti OR 'senium':ab,kw,ti OR 'elder':ab,kw,ti OR 'elder*':ab,kw,ti OR 'senior*':ab,kw,ti OR 'geriatric*':ab,kw,ti OR 'older adult*':ab,kw,ti OR 'older people':ab,kw,ti** | **#1 AND #2** | (*n* = 794; Mar 15, 2024) | (*n* = 1030; Nov 4, 2024) |
| **CINAHL** | **S1：****SU 'social frailty' OR SU 'social frail*' OR SU 'social vulnerability' OR SU 'social vulnerabilit*'**  **S2：(MH 'Aged') OR (SU** **elder OR SU** **elder* OR SU senior* OR SU geriatric* OR SU 'older adult*' OR SU 'older people')** | **S1 AND S2** | (*n* = 621; Mar 15, 2024) | (*n* = 656; Nov 4, 2024) |

**Table S2. Data charting form and items**

| **Key aspects** | **Items** | **Sub-items** |
| --- | --- | --- |
| **Theoretical framework** | Study | - Authors - Year - Place - Study design |
|  | Operational definition |  |
|  | Dimensions/subdimensions | - Number of dimensions/subdimensions - Details of each |
|  | Related SF measurement tools |  |
| **Measurement tools** | Study | - Authors - Year - Place - Study design - Population (study domain) |
|  | Instrument name |  |
|  | Theoretical framework |  |
|  | Dimensions | - Number of dimensions/subdimensions - Details of items or questions in each |
|  | Scoring structure | - Scale - Range - Scoring calculation - Cut-off for SF categorization |
|  | Interpretation |  |
| **Determinants** | Study | - Authors - Year - Place - Study design - Population (study domain) |
|  | Determinants | - Groups of determinants - Measurement tools - Data handling for analysis (e.g., categorization, input as a numerical score) |
|  | Instruments for SF | - Instrument name - Data handling for analysis (e.g., categorization, input as a numerical score) - Outcome assessors |
|  | Reported associations | - Outcome parameters (e.g., OR, RR, HR, β) - Methods of analysis - Confounder adjustment |
|  | Interpretation |  |
| **Consequence** | Study | - Authors - Year - Place - Study design - Population (study domain) |
|  | Instruments for SF | - Instrument name - Data handling for analysis (e.g., categorization, input as a numerical score) |
|  | Outcomes | - Groups of outcomes - Measurement tools - Data handling for analysis (e.g., categorization, input as a numerical score) - Outcome assessors |
|  | Reported associations | - Outcome parameters (e.g., OR, RR, HR, β) - Methods of analysis - Confounder adjustment |
|  | Interpretation |  |
| **Intervention** | Study | - Authors - Year - Place - Study design - Population (study domain) |
|  | Intervention | Types of interventions |
|  | Instruments for SF | - Instrument name - Data handling for analysis (e.g., categorization, input as a numerical score) - Outcome assessors |
|  | Efficacy | - Outcome parameters (e.g., OR, RR, HR, β) - Methods of analysis - Confounder adjustment |
|  | Interpretation |  |

**Table S3.** Summary of eligible articles for the review

| **Authors (Year), Place** | **Study type and design** | **Population and sample size** | **Key aspects** | | | | | **Variables** | |
| --- | --- | --- | --- | --- | --- | --- | --- | --- | --- |
|  |  |  | **Theoretical framework** | **Measurement tools** | **Determinants** | **Consequence** | **Intervention** | **Determinants**  **(Confounders)** | **Outcomes (Outcome parameter)** |
| Mitnitski et al.(2001), Canada[1] | Quantitative,  cross-sectional study | Older adults aged 65-106 years  (N = 2913) | Deficit accumulation model |  |  |  |  | Deficits accumulated model - Frail index (adjusted for age, patterns of cognitive impairments) | All-cause mortality |
| Steverink et al. (2006), Netherlands[2] | Quantitative,  cross-sectional study | Older adults aged 65-98 years  (N = 1322) | Social Production Function (SPF) theory |  |  |  |  | Levels of social need satisfaction, including, affection, behavioral confirmation, and status (adjusted for age, gender, physical losses) | Indicators of subjective well-being (life satisfaction, positive affect, negative affect) |
| Andrew et al.(2014), Canada[3] | Quantitative,  prospective cross-sectional study | Older adults aged 65 and above  (N = 2740) | Social Ecology Theory (SET) |  |  |  |  | Social frailty indicated by social vulnerability index adjusted for (age, sex,  educational attainment, frailty) | 10-year mortality  (adjusted HR) |
| Bunt et al. (2017), Netherlands [4] | Qualitative study  (scoping review) |  | Social Needs Fulfilment (SNF) theory |  |  |  |  | N/A | N/A |
| Pinsker et al.(2006), Australia[5] | Quantitative, cross-sectional study | Community-dwelling older adults aged ≥50 years (N=167) |  | 22-item Social Vulnerability Scale (SVS-22) |  |  |  | Social frailty indicated by SVS-22, with higher total scores indicating greater social frailty (Age, gender, educational attainment, income, social support, neurological condition） | Internal consistency and test-retest reliability of SVS-22 |
| Pinsker et al. (2011), Australia [6, 7] | Quantitative, cross-sectional study | Community-dwelling older adults aged ≥50 years  (N=266) |  | 15-item Social Vulnerability Scale (SVS-15) |  |  |  | Social frailty indicated by SVS-15, with higher total scores indicating greater social frailty (cognitive status, age, frequency of contact with the informant, relationship to informant) | Group differences in social vulnerability (SVS-15 total and subscale scores: gullibility and credulity) |
| Andrew et al.(2008), Canada [8] | Quantitative,  retrospective cohort study | 1.CSHA cohort: Community-dwelling adults aged ≥70 years, (N = 3707)  2. NPHS cohort: Community-dwelling adults aged ≥65 years, (N = 2648) |  | Social Vulnerability Index (SVI) |  | Mortality |  | Social frailty indicated by SVI constructed from 40 items in CSHA / 23 items in NPHS, with higher index value greater social frailty (adjusted for age, sex, and frailty index) | 5- to 8-year mortality  (adjusted OR) |
| Garre-Olmo et al.(2013), Spain[9] | Quantitative, prospective cohort study | Community-dwelling adults aged ≥74 years  (N = 875) |  | Social frailty phenotype (SFP） |  | Mortality |  | Social frailty indicated by SFP≥ 2 (adjusted for age, sex, marital status) | 4-year mortality  (adjusted HR) |
| Makizako et al. (2015),  Japan[10] | Quantitative, prospective cohort study | Community-dwelling Japanese adults aged ≥65 years  (N = 4304) |  | The Makizako Social Frailty Index (MSFI) of 5 items |  | Morbidity |  | Social frailty indicated by the Makizako Social Frailty Index of 5 items≥2 (adjusted for age, sex, BMI, MMSE, medications, hypertension, heart disease, diabetes, osteoporosis, GDS, and physical frailty status) | 2-year incidence of disability (adjusted HR) |
| Teo et al.(2017), Singapore[11] | Quantitative, cross-sectional and longitudinal analyses of a population-based cohort | Older adults aged ≥55 years; N = 2406 for baseline cross-sectional analysis；N = 1254/1557 for longitudinal IADL/severe disability analysis. |  | 7-item social frailty index (SFI-7） |  | Morbidity |  | Social frailty indicated by 7-item SFI≥2 (adjusted for age, sex, marital status, physical frailty, MMSE, depression, self-rated health) | Incident IADL disability and severe ADL disability (adjusted OR) |
| Ma et al. (2018), China[12] | Quantitative, prospective cohort study | Older adults aged ≥60 years  (N = 1697) |  | HALFT scale |  | Mortality |  | Social frailty indicated by HALFT≥3 | 8-year mortality  (adjusted HR) |
| Yamada et al.(2018), Japan[13] | Quantitative, prospective cohort study | Older adults aged ≥65 years  (N = 6603) |  | 4-item social frailty screening index |  | Morbidity |  | Social frailty indicated by 4-item social frailty index≥2 (adjusted for age, sex, BMI, medications, and comorbidities in Cox model; items: financial difficulty, living alone, no social activity, low neighbor contact) | 6-year incidence of disability or mortality (adjusted HR) |
| Yoo et al. (2019), Korea[14] | Quantitative, cross-sectional study | Older adults aged 70–84 years  (N = 1539) |  | Social frailty scale (SFS） |  |  |  | Social frailty indicated by SFS≥3(adjusted for age, residence, SES, depression, MMSE, physical frailty) | Prevalence of moderate hearing loss (PTA> 40 dB) and its association with social frailty  (adjusted OR) |
| Pek et al.(2020), Singapore[15] | Quantitative, cross-sectional study | Older adults aged ≥50 years  (N = 229) |  | 8-item social frailty scale (SFS-8) |  | Psychological and mental health |  | Social frailty indicated by SFS-8≥4 (adjusted for age, sex, education, hypertension, albumin, and physical frailty) | Poor mood (GDS≥4), poor physical performance (SPPB<10), low physical activity (IPAQ<2826 METs) (adjusted OR) |
| Chen et al. (2021), China[16] | Quantitative, cross-sectional and longitudinal study | Older adults aged 70–84 years.  The cross-sectional sample was N = 1764, of which N = 1174 were used for longitudinal analysis |  | 6-item social frailty scale (SFS-6) |  | Psychological and mental health |  | Social frailty indicated by 6-item scale≥4 (adjusted for age, sex, marital status, literacy, smoking, alcohol intake, BMI, and number of medical conditions) | 3-year incidence of depressive symptoms and worsening depressive symptoms  (adjusted OR) |
| Irshad et al.(2024), India[17] | Quantitative, cross-sectional study | Older adults aged ≥60 years  (N = 23,361) |  | Social Frailty  Index (SFI) | Cognitive functions,  Psychological and mental health |  |  | Low cognitive health status: a set of questions on memory, orientation, arithmetic function, executive function, and object naming. Scores range from 0-43; in this study, the lowest 10% has low cognitive status  (adjusted for age, sex, education, economic status, social group, rural/urban, social welfare, ADL, IADL, morbidity, self-rated health, depression, cognitive function) | Social frailty indicated by SFI, with higher scores indicates greater social frailty  (adjusted β-coefficient) |
| Inoue et al. (2022), Japan[18] | Quantitative, cross-sectional study | Older adults aged ≥65 years  (N = 495) |  |  | Physical function and physical frailty |  |  | Physical frailty by frailty phenotype: presence of three or more of shrinking, weakness, exhaustion, slowness, and low activity (adjusted for age, sex, BMI, MNA-SF, MMSE, physical frailty, osteoporosis medication, education level, number of comorbidities and medications) | Social frailty indicated by 4-item scale≥2  (adjusted OR) |
| Nagai et al. (2020),  Japan[19] | Quantitative, prospective cohort study | Older adults aged ≥65 years  (N = 342) |  |  | Physical function and physical frailty |  |  | 1. Physical frailty by frailty phenotype: presence of three or more of shrinking, weakness, exhaustion, slowness, and low activity  2.Gait speed slowness defined as a speed <1.0m/s  3.Hand grip strength: < 26kg for men, <18kg for women indicates strength weakness  (adjusted for age, sex, MMSE, GDS, multimorbidity, IADL, fall history) | 2-year incidence of social frailty indicated by modified 4-item SFI≥2  (adjusted RR) |
| Shega et al. (2012), Canada[20] | Quantitative, cross-sectional study | Older adults aged≥65 years  (N = 3,767) |  |  | Cognitive functions,  Pain |  |  | 1. Cognitive impairment: 3MS range of 0–100, with higher scores indicating better cognition. Scores less than 77 indicate cognitive impairment 2. Pain - moderate to severe: 5-point verbal descriptor scale  (adjusted for age, sex, ethnicity, education, comorbidity, depression, functional impairment via IADL) | Social frailty indicated by 39-item SVI, with higher scores indicate more vulnerable (adjusted β-coefficient) |
| Kodama et al. (2022), Japan[21] | Quantitative, prospective cohort study | Older adults aged≥65 years  (N = 103) |  |  | Psychological and mental health |  |  | Depression symptoms by GDS-15 score, higher scores indicate a higher risk of having depressive symptoms (adjusted for age, gender, education, medication number, walking speed, grip strength, cognitive tests, and GDS-15 score) | 1-year transition from robust to social frailty indicated by Makizako’s 5-item scale≥2  (adjusted OR） |
| Kume et al. (2022), Japan[22] | Quantitative, cross-sectional study | Older adults aged≥65 years  (N = 313) |  |  | Psychological and mental health |  |  | Depression symptoms by GDS-15 score, higher scores indicate a higher risk of having depressive symptoms (adjusted for age, gender, education, polypharmacy, hypertension, diabetes, hyperlipidemia) | Social frailty indicated by Makizako’s 5-item scale≥2 (adjusted OR) |
| Van Oostrom et al.(2017), Netherlands  [23] | Quantitative, cross-sectional study | Community-dwelling adults aged 40–81years (N = 4019) |  |  | Lifestyle factors |  |  | 1.Physically active  defined as 30 minutes of moderate to vigorous physical activity per day on at least 5 days per week  2. Sleep duration  ≤5 hours (adjusted for age, sex, education, marital status, living alone, work status, physical activity, smoking, sleep duration, multimorbidity, etc.) | Social frailty indicated by ≥2/3 criteria from (1) Loneliness Scale, (2) Social Support List-12, and (3) limited social participation  (adjusted OR) |
| Ye et al. (2021), Netherlands[24] | Quantitative, cross-sectional study | Older adults aged≥70 years  (N = 2289) |  |  | Lifestyle factors  Multimorbidity  Medication risk |  |  | 1. Physical activity  once a week or less  2.Multimorbidity  defined as having at least 2 of 14 common  chronic conditions  3. Medication risk by MRQ-10 scores: higher scores refer to a higher level of inappropriate medication use  (adjusted for age, sex, country, education, household composition, alcohol use risk, physical activity, multimorbidity, medication risk, malnutrition) | Social frailty indicated by TFI -3 items, cutoff ≥2 defines social frailty (adjusted OR) |
| Nakakubo et al. (2019), Japan[25] | Quantitative, cross-sectional study | Older adults aged≥65 years  (N = 4427) |  |  | Lifestyle factors (sleep problems) |  |  | 1.Long sleep duration  defined as ≥9.0 hours  2.EDS, Almost always have daytime sleepiness requiring a nap (adjusted for age, sex, BMI, education, medication, hypertension, heart disease, respiratory disease, diabetes, alcohol and smoking habits, physical activity, gait speed, MMSE, and depressive symptoms) | Social frailty indicated by Makizako’s 5-item scale≥2 (adjusted OR) |
| Henry et al. (2023), Australia[26] | Quantitative, cross-sectional study | Older adults aged≥65 years  (N = 90) |  |  | Lifestyle factors | Psychological and mental health |  | Social behavior by SDS score: higher scores indicate a greater social dysfunction (adjusted for physical frailty, cognitive frailty, depression) | Social frailty indicated by SFS-8  (adjusted β-coefficient) |
|  |  |  |  |  |  |  |  | Social frailty indicated by SFS-8 (adjusted for physical frailty, cognitive frailty, depression) | Psychological outcomes  1.Demoralization by DS: score range of 0–96, where higher scores indicate more severe demoralization  2.Resilience by 14-RSS: score range of 14–98, higher scores  indicated higher resilience  3.Satisfaction with life by SLS: score range of 5-35; higher scores indicate higher satisfaction  (adjusted β-coefficient) |
| Hirase et al. (2019), Japan[27] | Quantitative, cross-sectional study | Older adults aged≥65 years  (N = 248) |  |  | Pain |  |  | Chronic pain by rating scale score of ≥5 and related symptoms within the past month that had continued for at least 6 months (adjusted for age, sex, and physical function) | Social frailty indicated by 5-item Makizako scale≥ (adjusted OR) |
| Gobbens et al. (2024),  Netherlands[28] | Quantitative, cross-sectional study | Older adults aged≥65 years  (N = 24,347) |  |  | Multimorbidity |  |  | Multimorbidity: the combinations of diabetes mellitus, cancer, hypertension, arthrosis, urinary incontinence, and severe back disorder | Social frailty indicated by TFI ≥ 2 of 3 criteria  (adjusted β-coefficient) |
| Park et al. (2019), Korea[29] | Quantitative, cross-sectional study | Older adults aged≥65 years  (N = 408) |  |  |  | Morbidity,  Psychological and mental health |  | Social frailty indicated by Makizako’s 5-item questionnaire≥2 (adjusted for age, sex, and physical frailty as per CHS scale) | 1.ADL disability defined as requiring assistance in any ADL  2. Depressed mood by Korean CES-D: ≥21  (adjusted OR) |
| Doi et al. (2022), Japan[30] | Quantitative, prospective cohort study | Older adults aged≥65 years  (N = 4642) |  |  |  | Morbidity |  | Social frailty indicated by 5-item Makizako scale≥2 (adjusted for age, sex, hypertension, hyperlipidemia, diabetes, number of medications, physical inactivity, MMSE score) | 5-year disability incidence from long-term care insurance system  (adjusted HR) |
| Doi et al. (2024), Japan[31] | Quantitative, prospective cohort study | Older adults aged≥65 years  (N = 8,301) |  |  |  | Morbidity |  | Social frailty indicated by 5-item Makizako scale≥2 (adjusted for age, sex, chronic diseases, number of medications, education, depressive symptoms [GDS], cognitive impairment [MMSE], AMI） | 2-year disability incidence from long-term care insurance system  (adjusted HR) |
| Cooper et al. (2022),  Tanzania[32] | Quantitative, prospective cohort study | Community-dwelling older adults aged≥60 years  (N = 235) |  |  |  | Mortality |  | Social frailty indicated by SVI derived from 48 culturally adapted items (adjusted for age and sex in Model 1; additionally adjusted for Frailty Index in Model 2) | Mortality  (adjusted HR) |
| Sun et al. (2023), China  [33] | Quantitative, prospective cohort study | Older adults aged≥65 years  (N = 460) |  |  |  | Mortality |  | Social frailty indicated by modified 4-item index≥2（adjusted for age, sex, education, marital status, sleep quality, cognition, depression） | 3 to 6-year mortality (adjusted OR) |
| Makizako et al. (2018),  Japan[34] | Quantitative, prospective cohort study | Older adults aged≥65 years  (N = 1226) |  |  |  | Physical function and physical frailty |  | Social frailty indicated by 5-item scale≥2 (adjusted for age, gender, BMI, MMSE, medication count, hypertension, heart disease, diabetes, osteoporosis, grip strength, walking speed) | 4-year incidence of physical frailty and physical pre-frailty (adjusted OR) |
| Makizako et al. (2019), Japan[35] | Quantitative, cross-sectional study | Older adults aged≥65 years  (N = 353) |  |  |  | Physical function and physical frailty |  | Social frailty indicated by Makizako’s 5-item scale≥2  （adjusted for age, education, medication count, and physical activity） | Muscle weakness  (adjusted OR) |
| Huang et al. (2021), Japan[36] | Quantitative, prospective cohort study | Older adults aged≥60 years  (N = 663) |  |  |  | Physical function and physical frailty,  Cognitive functions,  Psychological and mental health |  | Social frailty indicated by 4-item score≥2 (adjusted for age, sex, education, BMI, Charlson Comorbidity Index (CCI), physical activity) | 3-year change in intrinsic capacity composite score (adjusted β-coefficient) |
| Armstrong et al. (2015), Canada[37] | Quantitative, prospective cohort study | Older men aged 71–93 years  (N = 3845) |  |  |  | Cognitive functions |  | Social frailty indicated by SVI, with higher indicates worse social vulnerability (adjusted for age (centered at 78), education (centered at 10.5 years), frailty index) | Cognitive decline over 3 and 6 years  (adjusted β-coefficient) |
| Zhang et al. (2024), China[38] | Quantitative, prospective cohort study | Older adults aged≥65 years  (N = 7732) |  |  |  | Cognitive functions |  | Social frailty indicated by Makizako 5-item index≥2  (adjusted for age, sex, race, education, BMI, smoking, sleep disorders, physical activity, multimorbidity, depressive and anxiety symptoms) | Incident MCR (motoric cognitive risk syndrome) over median 4 years  (adjusted HR) |
| Hayashi et al.(2022), Japan[39] | Quantitative, cross-sectional study | Older adults aged≥65 years  (N = 1103) |  |  |  | Psychological and mental health |  | Social frailty indicated by 4-item SFSI≥2 (adjusted for age, sex, BADL, IADL, education, physical activity, comorbidities, and exercise habits at home) | Depressive symptoms（adjusted OR） |
| Ko et al.(2021), Korea[40] | Quantitative, cross-sectional study | Older adults aged≥65 years  (N = 10,081) |  |  |  | Psychological and mental health |  | Social frailty indicated by 5-item scale≥2 (adjusted for sociodemographic variables, number of chronic diseases, prescribed medications, physical activity, subjective health, cognitive function, depression, nutrition) | Depression and life Satisfaction  (adjusted β-coefficient) |
| Noguchi et al.(2021), Japan[41] | Quantitative, cross-sectional study | Older adults aged≥60 years  (N = 300) |  |  |  | Psychological and mental health |  | Social frailty indicated by 5-item Makizako scale≥2（adjusted for age, gender, education, income, employment, present illness, IADL, BMI, drinking, smoking, frequent urination） | Sleep quality  (adjusted β-coefficient) |
| Pollak et al.(2022), America[42] | Randomized Controlled Trial (RCT) | Older adults aged≥65 years  (N=220,intervention group=107; control group=113) |  |  |  |  | Robotic pet | Social frailty indicated by 5-item Makizako scale≥2 (adjusted for age, sex, race, education, marital status, living arrangement, depression, cognition) | Social frailty status  (adjusted mean difference) |

**Abbreviations:** SF, Social Frailty;SET, Social Ecology Theory; SPF, Social Production Function theory; SNF, Social Needs Fulfilment theory;SVS, Social Vulnerability Scale; CSHA, the Canadian Study of Health and Aging; NPHS, the National Population Health Survey; SVI, Social Vulnerability Index;OR, Odds Ratio;MSFI, The Makizako Social Frailty Index;SFP, Social Frailty Phenotype;HR, Hazard Ratio;IADL, Instrumental Activities of Daily LivingADL, Activity Daily Living Index;BMI,Body Mass Index;MMSE,Mini-Mental State Examination;GDS, Geriatric Depression Scale;SFI, Social Frailty Index;SFS,social frailty scale;PTA,Pure-tone average;MNA-SF, Mini-Nutritional Assessment-short form;CHS,The Cardiovascular Health Study;TFI, the Tilburg Frailty Indicator; AMI, Active Mobility Index; CCI,Charlson Comorbidity Index;MCR, Motoric Cognitive Risk Syndrome;BADL,Basic Activities of Daily Living.

**Table S4.** Summary of excluded articles for the review

| **Authors (Year), Place** | **Study type and design** | **Population**  **and sample size** | **Key aspects** | | | | | **Reasons to exclude** |
| --- | --- | --- | --- | --- | --- | --- | --- | --- |
|  |  |  | **Theoretical framework** | **Measurement tools** | **Determinants** | **Consequence** | **Intervention** |  |
| Hilt et al. (2018), USA[43] | Quantitative, retrospective cohort study | Adult patients from inpatient geriatric co-management services  (N=214) |  |  |  | 1.hospital readmission  2.mortality |  | Conference abstract |
| Ma et al. (2019), China[44] | Quantitative, retrospective cohort study | Community-dwelling old adults aged ≥ 60 years from Beijing Longitudinal Study of Aging  (N=1,697) |  |  |  | Mortality |  | Conference abstract |
| Osokpo et al. (2022), USA[45] | Quantitative, cross-sectional study | Adults aged ≥ 35 years with chronic disease  (N=128) |  |  |  | Lower self-care monitoring |  | Conference abstract |
| Shahid et al. (2023), USA[46] | Quantitative, retrospective cohort study | Adults ≥18 years with at least 1 outpatient visit from Houston Methodist Learning Health System Registry  (N=983,497) |  |  |  | Major adverse cardiovascular events |  | Conference abstract |
| Lim et al. (2023), Singapore[47] | Quantitative, prospective cohort study | Community-dwelling old adults  (N=151) |  |  | Pandemic control measures |  |  | Conference abstract |
| Fowler et al. (2023), USA[48] | Quantitative, retrospective cohort study | Older adults ≥ 60 years  (N=645) |  |  |  | 1.geriatric assessments  Impairment  2.frailty |  | Conference abstract |
| Jain et al. (2022), USA[49] | Quantitative, retrospective cohort study | Adults ≥18 years from the Behavioral Risk Factor Surveillance System 2016 to 2019  (N=1,745,999) |  |  | Cardiovascular Diseases (including diabetes, hypertension, hyperlipidemia, smoking, substance use) |  |  | Wrong population  (Adults ≥18 years) |
| Neiman et al. (2022), USA[50] | Quantitative, retrospective cohort study | Adults with trauma admissions from January 2017 to September 2020  (N = 83,607) |  |  |  | Trauma mortality |  | Wrong population  (Adults with trauma) |
| Miao et al. (2022), China[51] | Quantitative, retrospective cohort study | Middle-aged and older adults with chronic diseases  (N=4,097) |  |  |  | Catastrophic health expenditure |  | Wrong population  (involved both middle-aged and older adults with chronic diseases) |
| Wang et al. (2023), China[52] | Quantitative, cross-sectional study | Older patients with chronic heart failure  (N=205) |  |  | Social support |  |  | Wrong population  (older patients with chronic heart failure) |
| Chen et al. (2023), USA[53] | Quantitative, cross-sectional study | All cancer-related deaths across US counties from 2013 to 2019 (N=107,273) |  |  |  | Cancer-Related Mortality |  | Wrong population (cancer patients) |
| Pascal et al. (2004), French[54] | Quantitative, cross-sectional study | Adults aged from 15 to 93 years  (N=222) |  | five characteristics identifying social vulnerability, integrated in a self-reported questionnaire |  |  |  | Articles not written in English |
| Díaz-Alonso et al. (2021), Spain[55] | Quantitative, cross-sectional study | Non-institutionalized adults aged ≥ 65 years  (N=445) |  |  | Hearing loss |  |  | Articles not written in English |
| dos Santos et al. (2022), Brazil[56] | Quantitative, retrospective cohort study | COVID-19 patients aged ≥ 50 years  (N=410,504) |  |  |  |  | Survival and hospital lethality | Articles not written in English |
| Nagano et al. (2022), Japan[57] | Quantitative, cross-sectional study | Older adults  (N=171) |  | Questionnaire for the screening of frailty by the Japan Geriatrics  Society  (15 questions) |  |  |  | Articles not written in English |
| Tarazona-Santabalbina et al. (2016), Spain[58] | Quantitative, RCT | Frail older adults (N=100) |  |  |  |  | Supervised-facility multicomponent exercise program | Not include social frailty as a study variable |
| Verver et al. (2019), Netherlands[59] | Quantitative, cross-sectional study | Community-dwelling older adults aged ≥ 65 years  (N=1,768) |  |  |  | 1.health outcomes 2.well-being aspects for independent living |  | Not include social frailty as a study variable (measured Tilburg Frailty Index as a multicomponent frailty) |
| Aznar-Tortonda et al. (2020), Spain[60] | Quantitative, cross-sectional study | Older adults (N=761) |  |  |  |  |  | Not include social frailty as a study variable |
| Hoogendijk et al. (2020), Netherlands[61] | Quantitative, prospective cohort study. | Community-dwelling older adults aged ≥ 65 years  (N=1,427) |  |  |  | Mortality |  | Not include social frailty as a study variable (measured only “Loneliness” and “Social isolation”) |
| Gelmini et al. (2020), Italy[62] | Quantitative, cross-sectional study | Older adults (N=761) |  |  | 1.logistic and housing conditions 2.disease 3.medications 4.basic and instrumental ADL 5.neurological 6.psychological conditions. |  |  | Not include social frailty as a study variable |
| Harada et al. (2021), Japan[63] | Quantitative, non-RCT | Community-dwelling older adults aged ≥ 60 years  (N=662) |  |  |  |  | Neighborhood social network intervention (3 years) | Not include social frailty as a study variable |
| De Luca et al. (2021), Italy[64] | Quantitative, RCT | Frail older adults (N=60) |  |  |  |  | Telemedicine | Not include social frailty as a study variable |
| Papathanasiou et al. (2021),  Greece[65] | Quantitative, cross-sectional study | Older adults aged between 61 and 96 years (N=257) |  |  | Multimorbidity (≥ 2 chronic diseases), traumatic events |  |  | Not include social frailty as a study variable (measured Tilburg Frailty Index as a multicomponent frailty) |
| Melchiorre et al. (2022), Italy[66] | Qualitative, cross-sectional study | Community-dwelling older adults aged ≥ 65 years  (N=120) |  |  |  | 1.health emergencies 2.falls  3.use of communication technologies |  | Not include social frailty as a study variable (no standard tool was used to assess frailty) |
| Roth et al. (2022), USA[67] | Quantitative, retrospective, cohort study | COVID-19 patients aged ≥ 18 years from the Providence health system electronic health record  (N=11,326) |  |  |  | Outpatient Health Care Utilization |  | Not include social frailty as a study variable (measured community-level Social Vulnerability Index) |
| Millan-Domingo et al. (2022), Spain[68] | Quantitative, pragmatic trials | Frail older adults aged ≥ 70 years (N=50) |  |  |  |  | Multicomponent exercise intervention | Not include social frailty as a study variable |
| Satsanasupint et al. (2022), Thailand[69] | Qualitative, cross-sectional study | Community-dwelling older adults aged ≥ 60 years  (N=20) |  |  |  |  | Community networks | Not include social frailty as a study variable |
| Faria et al. (2022), Portugal[70] | Quantitative, cross-sectional study | Older adults ≥ 65 years registered at a Health Care Unit in Northern Portugal (N=300) |  |  |  | Individual Lifestyle |  | Not include social frailty as a study variable (measured Tilburg Frailty Index as a multicomponent frailty) |
| Jacobs et al. (2023), USA[71] | Quantitative, cross-sectional study | Older adults ≥ 65 years from the Behavioral Risk Factor Surveillance System years  (N= 30,648) |  |  |  | Individual physical and mental wellness |  | Not include social frailty as a study variable (measured community-level Social Vulnerability Index) |
| Ohta et al. (2024), Japan[72] | Quantitative,  Non-RCT | Community-dwelling older adults aged ≥ 65 years  (N=34) |  |  |  |  | mHealth application “Online Kayoinoba”  (designed to enhance physical activity duration and social interaction opportunities for seniors during COVID-19) | Not include social frailty as a study variable |

**Abbreviations:** ADL, Activity daily living**;** RCT, Randomized Control Trial.

**Table S5. Determinants associated with Social Frailty**

| **Determinants** | **Instruments for SF** | **Reported associations** | **Interpretation** | **Reference** |
| --- | --- | --- | --- | --- |
| **Physical function and physical frailty** | | | | |
| Physical frailty  (frailty phenotype: presence of three or more of shrinking, weakness, exhaustion, slowness, and low activity) | 4-item SFSI  (≥2 indicates social frailty) | aOR2.126  (95%CI 1.432 to 3.155)  ^*adjusted for age, sex, BMI, education, medication, MNA-SF, and MMSE^ | Physical frailty was significantly associated with a higher risk of turning into social frailty. | Inoue,  2022,  (Japan)[18] |
| Physical frailty  (frailty phenotype: presence of three or more of shrinking, weakness, exhaustion, slowness, and low activity) | 4-item  SFSI(≥2 indicate social frailty)  (social frailty assessed by two domains: social activity and contact with neighbors) | At 2 years  aRR 1.78  (95%CI1.10-10.53)  ^*adjusted for age, sex, GDS, MMSE, multimorbidity, fall, and IADL^ | Physical frailty was significantly associated with a higher risk of turning into social frailty. | Nagai,  2020,  (Japan)[19] |
| Gait speed  (slowness is defined as a speed＜1.0m/s) | 4-item  SFSI(≥2 indicate social frailty)  (social frailty assessed by two domains: social activity and contact with neighbors) | At 2 years  aRR3.41  (95%CI 1.10-10.53)  ^*adjusted for age, sex, GDS, MMSE, multimorbidity, fall, and IADL^ | Slower walking speed was significantly associated with turning into social frailty. | Nagai,  2020,  (Japan)[19] |
| Muscle strength  (Hand grip strength: ＜26kg for men,＜18kg for women indicates strength weakness) | 4-item  SFSI (≥2 indicates social frailty)  (social frailty assessed by two domains: social activity and contact with neighbors) | At 2 years  aRR1.06  (95%CI 1.01-1.12)  ^*adjusted for age, sex, GDS, MMSE, multimorbidity, fall, and IADL^ | Weaker muscle strength was significantly associated with turning into social frailty. | Nagai,  2020,  (Japan)[19] |
| **Cognitive functions** |  |  |  |  |
| Cognitive impairment  (3MS range of 0–100, with higher scores indicating better cognition. Scores less than 77 indicate cognitive impairment) | SVI  (39 self-reported variables, range 0-1) | β0.49  (95% CI 0.13 to 0.86)  ^*adjusted for age, female, caucasian, education, comorbidity, depression and IADL^ | Cognitive impairment was associated with turning into social frailty. | Shega,  2012,  (Canada)[20] |
| Low cognitive health status  (a set of questions on memory, orientation, arithmetic function, executive function, and object naming. Scores range from 0-43; in this study, the lowest 10% has low cognitive status) | SFI  (score ranging from 0 to 100; higher score indicates a high level of social frailty) | β 3.17  (95%CI 2.21 to 4.13) | Low cognitive health status was significantly associated with turning into social frailty. | Irshad,  2024,  (India)[17] |
| **Psychological and mental health** | | | | |
| Depression  (GDS-15 score: higher scores indicate a higher risk of having depressive symptoms) | MSFI  (≥2 indicates social frailty) | aOR 1.57  (95%CI 1.15 to 2.13)  ^*adjusted for age, gender, medication, education, usual walk speed, grip strength, and cognitive subtests^ | A higher risk of having depressive symptoms was significantly associated with turning into social frailty. | Kodama, 2022, (Japan) [21] |
| Depression  (GDS-15 score: higher scores indicate a higher risk of having depressive symptoms) | MSFI  (≥2 indicates social frailty) | aOR 1.33  (95%CI 1.19 to 1.49)  ^*adjusted for age, gender, polypharmacy, education, hypertension, diabetes, dyslipidemia, and Trail Making Test A^ | A higher risk of having depressive symptoms was significantly associated with turning into social frailty. | Kume, 2022, (Japan) [22] |
| Depression  (CESD-10 score:≥4 indicate depression) | SFI  (score ranging from 0 to 100; higher score indicates a high level of social frailty) | β 1.63  (95%CI 0.83 to 2.44) | Depression was significantly associated with turning into social frailty. | Irshad,  2024,  (India)[17] |
| **Lifestyle factors** | | | | |
| Physically active  (defined as 30 minutes of moderate to vigorous physical activity per day on at least 5 days per week) | TFI  (≥2 of 3criteria indicate social frailty) | aOR 0.60  (95%CI 0.41to 0.86)  ^*adjusted for socio-demographic variables, lifestyle, life-events, biological risk factors, and chronic disease.^ | Being physically active was associated with a lower risk of social frailty. | van Oostrom,  2017,  (The  Netherlands)  [23] |
| Physical activity  (once a week or less) | TFI  (≥2 of 3criteria indicate social frailty) | Unadjusted OR1.51  (95%CI 1.17to 2.00)  reference: more than once a week | Physical activity once a week or less is associated with a higher risk of social frailty. | Ye,  2021,  (The Netherlands)  [24] |
| Sleep duration  (≤5hours) | TFI  (≥2 of 3criteria indicate social frailty) | aOR 3.11  (95%CI 1.73to 5.60)  reference: sleep duration(7to 8 hours)  ^*adjusted for socio-demographic variables, lifestyle, life-events, biological risk factors, and chronic disease.^ | Shorter sleep duration was associated with a higher risk of social frailty. | van Oostrom,  2017,  (The  Netherlands)  [23] |
| Long sleep duration  (defined as ≥9.0 hours) | MSFI  (≥2 indicates social frailty) | aOR1.75  (95%CI 1.29-2.38)  reference: sleep duration 6.1-8.9 hours  ^*adjusted for age, sex, BMI, education, medication, medical history, current drinking habit, current smoking habit, physical activity, gait speed, and MMSE.^ | Longer sleep duration was associated with a higher risk of social frailty. | Nakakubo,  2019,  (Japan)  [25] |
| EDS  (Almost always have daytime sleepiness requiring a nap) | MSFI  (≥2 indicates social frailty) | aOR1.48  (95%CI 1.07-2.06)  ^*adjusted for age, sex, BMI, education, medication, medical history, current drinking habit, current smoking habit, physical activity, gait speed, and MMSE.^ | Excessive daytime sleepiness was associated with a higher risk of social frailty. | Nakakubo,  2019,  (Japan)  [25] |
| Social behavior  (SDS score: higher scores indicate a greater social dysfunction) | SFS  (range 0-8) | β 0.24  (95%CI 0.04 to 0.44)  ^*controlling for physical frailty, cognitive frailty, and depression^ | Inappropriate social behavior was predictive of increased social frailty. | Henry,  2023,  (Australia)  [26] |
| **Other health indicators** | | | | |
| Pain (moderate to severe)  (5-point verbal descriptor scale) | SVI  (39 self-reported variables, range 0-1) | β0.44  (95% CI 0.21 to 0.66)  ^*adjusted for age, female, caucasian, education, comorbidity, depression and IADL^ | Moderate to severe pain has a moderate effect on developing social frailty. | Shega,  2012,  (Canada)[20] |
| Chronic pain  (the maximum pain reached a numerical rating scale score of ≥5 and related symptoms within the past month that had continued for at least 6 months) | MSFI  (≥2 indicates social frailty) | aOR2.13  (95%CI 1.01-4.48)  ^*adjusted for age, sex,^ ^and physical function measures.^ | A higher score of chronic pain was significantly associated with turning into social frailty. | Hirase,  2019,  (Japan)[27] |
| Multimorbidity  (Defined as having at least 2 of 14 common  chronic conditions) | TFI  (≥2 of 3criteria indicate social frailty) | Unadjusted OR1.75  (95%CI 1.06to 2.88) | Multimorbidity is associated with a higher risk of social frailty. | Ye,  2021,  (Netherlands)  [24] |
| Multimorbidity  (the combinations of diabetes mellitus, cancer, hypertension, arthrosis, urinary incontinence, and severe back disorder) | TFI  (≥2 of 3criteria indicate social frailty) | β 0.059  (*p*＜0.001) | Multimorbidity has a very weak effect on developing social frailty. | Gobbens,  2024,  Netherlands[28] |
| Medication risk  (MRQ-10 scores: higher scores refer to a higher level of inappropriate medication use) | TFI  (≥2 of 3criteria indicate social frailty) | Unadjusted OR1.1  (95%CI 1.03to 1.19) | A higher risk of medication is associated with a higher risk of social frailty. | Ye,  2021,  (Netherlands)  [24] |

**Abbreviations:** SF, Social Frailty; 4-item SFSI, 4-item Social Frailty Screening index; aOR, adjusted Odds Ratio; aRR, adjusted Relative Risk; CI, confidence Interval; 3MS, the Modified Mini-Mental Status Examination; SVI, Social Vulnerability Index; SFI, Social Frailty Index; GDS, Geriatric Depression Scale; CESD, the Center for Epidemiological Studies Depression; TFI, the Tilburg Frailty Indicator; MSFI, The Makizako Social Frailty Index; EDS, excessive daytime sleepiness; SFS, Social Frailty Scale; SDS, Socioemotional Dysfunction Scale; MRQ, the Medication Risk Questionnaire.

**Table S6.** **Consequence of Social Frailty**

| **Consequences** | **Instruments for SF** | **Reported associations** | **Interpretation** | **Reference** |
| --- | --- | --- | --- | --- |
| **Morbidity and mortality** | | | | |
| Disability (long-term care insurance system) | MSFI  (≥2 indicates social frailty) | at 2 years  aHR 1.66  (95%CI 1.54 to 1.90)  reference: non-social frailty  ^*adjusted for age, gender, BMI, medication, number of prescribed medications, hypertension, heart disease, diabetes mellitus, osteoporosis, GDS, and physical frailty status^ | Social frailty significantly increased the risk of future disability among community-dwelling older people. | Makizako,  2015 (Japan)  [10] |
| Severe dependency  (≥3 BADL) | 7-item SFI  (≥2 indicates social frailty) | Unadjusted OR 6.27  (95%CI 2.58 to 15.23)  reference: non-social frailty | Social frailty might be associated with severe dependency. | Teo,  2017 (Singapore) [11] |
| ADL disability (defined as requiring assistance in any ADL) | MSFI  (≥2 indicates social frailty) | aOR 2.54  (95%CI 1.26 to 5.13)  reference: non-social frailty  ^*adjusted for age, gender, and low income^  C-statistic 0.71 for predicting ADL disability using MSFI ≥1  (95%CI 0.65-0.77) | Social frailty was associated with requiring assistance in carrying out ADL and could be used as a screening tool for predicting ADL disability. | Park,  2019  (Korea)  [29] |
| Disability | MSFI  (≥2 indicates social frailty) | at 5 years  aHR 1.40  (95% CI 1.17 to 1.67)  reference: non-social frailty  ^*adjusted for age, gender, hypertension, hyperlipidemia, diabetes, medication numbers, physical inactivity, MMSE, and driving status^ | Social frailty was independently associated with disability. | Doi,  2022 (Japan) [30] |
| Disability (long-term care insurance system) or mortality | 4-item SFSI  (≥2 indicates social frailty) | at 6 years  aHR 1.71  (95%CI 1.54 to 1.90)  reference: non-social frailty  ^*adjusted for age, gender, BMI, medication, and comorbidities^ | Social frailty significantly increased an incidence of disability and mortality. | Yamada,  2018  (Japan)  [13] |
| Disability (long-term care insurance system) or mortality | MSFI  (≥2 indicates social frailty) | at 2 years  aHR 1.33  (95% CI 1.02 to 1.74)  reference: non-social frailty  ^*adjusted for active mobility index, age, gender, education, medication, hypertension, diabetes, hyperlipidemia, depressive symptoms, cognitive impairment, and physical inactivity^ | Social frailty was significantly associated with the occurrence of disability. | Doi,  2024 (Japan) [31] |
| Mortality | SVI  (range 0-1) | at 5 years (CSHA cohort)  aHR 1.03  (95%CI 1.01–1.05)  median (IQR) SVI 0.25 (0.20-0.31)  at 8 years (NPHS cohort)  aHR 1.04  (95%CI 1.01–1.07)  median (IQR) SVI 0.28 (0.21-0.35)  ^*adjusted for age, gender, and frailty^ | Social vulnerability was associated with higher mortality, independent of frailty. | Andrew, 2008 (Canada) [8] |
| Mortality | SFP  (≥ 2 phenotypes indicate social frailty) | at 4 years  aHR 2.69  (95%CI 1.01 to 7.25)  reference: non-social frailty  ^*adjusted for age, gender, marital status, physical frailty phenotype, and mental frailty phenotype^ | Social frailty significantly increased mortality risk among community-dwelling older people. | Garre-Olmo, 2013 (Spain) [9] |
| Mortality | HALFT scale  (≥1 indicates pre- and social frailty group) | at 8 years  aHR 1.91  (95%CI 1.54 to 2.33)  reference: non-social frailty  ^*adjusted for age and gender^ | Social frailty in older adults was significantly associated with lower survivability. | Ma, 2018  (China)  [12] |
| Mortality | SVI  (range 0-1) | at 1 years  aHR 1.13  (95%CI 1.05 to 1.22)  median (IQR) SVI 0.47 (0.14-0.86)  ^*adjusted for age and gender^ | Social frailty was significantly associated with mortality. An increase in SVI corresponds to a greater mortality risk. | Cooper, 2022  (Tanzania)  [32] |
| Mortality | 4-item SFSI  (≥2 indicates social frailty) | at 6 years  aOR 2.22  (95%CI 1.15 to 4.28)  reference: non-social frailty  ^*adjusted for age, self-reported memory status, and living with a confidant^ | Baseline social frailty was significantly associated with an increased mortality risk at 6 years. | Sun,  2023 (China)  [33] |
| **Physical function and physical frailty** | | | | |
| Physical frailty | MSFI  (≥2 indicates social frailty) | at 4 years  aOR 3.93  (95% CI 1.02 to 15.15)  reference: non-social frailty  ^*adjusted for age, gender, BMI, number of prescribed medications, hypertension, heart disease, diabetes mellitus, osteoporosis, grip strength (baseline) and walking speed (baseline)^ | Socially frailty older adults were at risk for developing physical frailty than non-socially frailty individuals. | Makizako,  2018  (Japan)  [34] |
| Muscle weakness  (Hand grip strength: ＜26kg for men,＜18kg for women indicates strength weakness) | MSFI  (≥2 indicates social frailty) | aOR2.04  (95%CI 1.06-3.95)  ^*adjusted for age, education, number of prescribed medications, and physical activity levels (>5 days/week).^ | Social frailty was significantly associated with muscle weakness. | Makizako,  2019,  (Japan)[35] |
| Vitality score  (Hand grip strength and MNA: transformed into a Z score) | 4-item SFSI  (≥2 indicates social frailty) | at 3 years  β -0.13  (95% CI -0.235 to -0.02)  ^*adjusted for age, educational level, BMI, CCI score, and BAQ score^ | Social frailty was significantly associated with physical function decline. | Huang,  2021 (Japan)  [36] |
| **Cognitive functions** | | | | |
| Cognitive decline  (Cognitive Abilities Screening Instrument 100-point scale: lower scores represent better cognition) | SVI  (range 0-1) | at 3 years  β 0.16  (95% CI 0.09 to 0.23)  at 6 years  β 0.14  (95% CI 0.05 to 0.24)  ^*adjusted for baseline cognitive state, FI, PI, age, and education^ | High SVI in older adults was associated with an increased risk of cognitive decline at 3 and 6 years. | Armstrong, 2015,  (Canada)  [37] |
| Cognitive functions (the five cognitive tests: transformed into a Z score) | 4-item SFSI  (≥2 indicates social frailty) | at 3 years (male)  β -0.51  (95% CI -0.68 to -0.34)  reference: non-social frailty  ^*adjusted for age, educational level, BMI, CCI score, and BAQ score^ | Male older adults with social frailty had an increased risk of cognitive impairment. | Huang,  2021 (Japan)  [36] |
| Motoric cognitive risk syndrome(MCR)  (defined as the presence of both subjective cognitive  complaints (without dementia) and slow gait speed) | MSFI  (≥2 indicates social frailty) | HR 1.57  (95%CI 1.34 to 1.84)  At 4 years  ^*adjusted for age, gender, race, educational level, smoking status, sleep disorders, body mass index, vigorous exercise, chronic diseases, depressive, and anxiety symptom^ | Older adults with social frailty had a higher risk of incident MCR | Zhang,  2024(China)[38] |
| **Psychological and mental health** | | | | |
| Depression  (Korean CES-D: ≥21 was classified as having a depressive mood) | MSFI  (≥2 indicates social frailty) | aOR 4.26  (95%CI 1.38 to 13.19)  reference: non-social frailty  ^*adjusted for age, gender, and low income^ | Social frailty has independently increased the risk of depressed mood. | Park,  2019  (Korea)  [29] |
| Depression  (GDS-15: ≥4 having depressive symptoms) | 8-item SFS  (≥4 indicates social frailty) | aOR 6.32  (95%CI 1.12 to 4.89)  reference: non-social frailty  ^*adjusted for age and gender^ | Social frailty was significantly associated with the presence of depressive symptoms. | Pek,  2020  (Singapore)  [15] |
| Depression  (GDS-15: ≥6 having depressive symptoms) | 6-item SFS  (≥4 indicates social frailty) | Incident depressive symptoms  aOR 2.31  (95%CI 1.10 to 4.88)  Worsening depressive symptoms  aOR 2.07  (95%CI 1.18 to 3.65)  reference: non-social frailty  ^*adjusted for age, gender, marital status, education, smoking, alcohol intake, BMI, and number of medications.^ | Social frailty increased the incidence of depressive symptoms and was associated with an increased risk of worsening  depressive symptoms. | Chen,  2021 (China) [16] |
| Depression  (GDS-15 score: transformed into a Z score) | 4-item SFSI  (≥2 indicates social frailty) | at 3 years  β -0.67  (95% CI -0.93 to -0.40)  ^*adjusted for age, educational level, BMI, CCI score, and BAQ score^ | Older adults with social frailty had an increased risk of having more depressive symptoms. | Huang,  2021 (Japan)  [36] |
| Depression  (K6 scale: ≥5 was classified as having depressive symptoms) | 4-item SFSI  (≥2 indicates social frailty) | aOR 1.80  (95%CI 1.16 to 2.79)  reference: non-social frailty  ^*adjusted for age, gender, BADL, IADL, low, non-exercise, low education, and comorbidity^ | Social frailty was significantly associated with depressive symptoms. | Hayashi,  2022  (Japan)  [39] |
| Demoralization  (DS: score range of 0–96, where higher scores indicate more severe demoralization) | SFS  (range 0-8) | β 0.33  (95%CI 0.17 to 0.49)  ^*controlling for physical frailty, cognitive frailty, and depression^ | Older adults with social frailty had an increased risk of having demoralization. | Henry,  2023,  (Australia)  [26] |
| Resilience (14-RSS: score range of 14–98, higher scores  indicated higher resilience） | SFS  (range 0-8) | β 0.27  (95%CI -0.47 to -0.08)  ^*controlling for physical frailty, cognitive frailty, and depression^ | Social frailty is associated with lower psychological resilience. | Henry,  2023,  (Australia)  [26] |
| Satisfaction with life (SLS: score range of 5-35; higher scores indicate higher satisfaction) | SFS  (range 0-8) | β -0.47  (95%CI -0.64 to -0.30)  ^*controlling for physical frailty, cognitive frailty, and depression^ | Older adults with social frailty have lower life satisfaction. | Henry,  2023,  (Australia)  [26] |
| Life Satisfaction  (Questions about 6 aspects of life, score range of 6-30; higher scores indicated higher levels of life satisfaction) | MSFI  (≥2 indicates social frailty) | β-0.267  (*p*＜0.001) | Older adults with social frailty tend to have lower life satisfaction. | Ko,  2021,  (Korea)  [40] |
| Sleep Quality  (PSQI score) | MSFI  (≥2 indicates social frailty) | β 0.93(95% CI 0.09 to 0.23)  reference: non-social frailty  ^*adjusted for age, gender, education, income, employment status, present illness, instrumental ADL, body mass index, drinking, smoking, and frequent urination^ | Social frailty was significantly associated with poor sleep quality. | Noguchi,  2021  (Japan)  [41] |

**Abbreviations:** SF, Social Frailty; MSFI, The Makizako Social Frailty Index; HR, Hazard Ratio; CI, confidence Interval; BADL, Basic Activity Daily Living Index; SFI, Social Frailty Index; aOR, adjusted Odds Ratio; ADL, Activity Daily Living Index; SFSI, Social Frailty Screening index; SVI, Social Vulnerability Index; CSHA, the Canadian Study of Health and Aging; NPHS, the National Population Health Survey; IQR, Interquartile Range; SFP, Social Frailty Phenotype; MNA, Mini-Nutritional Assessment; MCR, Motoric Cognitive Risk Syndrome; Korean CES-D, the Korean version of the Center for Epidemiological Studies Depression scale; GDS, Geriatric Depression Scale; SFS, Social Frailty Scale; K6, Kessler 6 scale; DS: the Demoralization Scale; RSS: the short-form Resilience Scale; SLS: the Satisfaction with Life Scale; PSQI, Pittsburgh Sleep Quality Index.

**References**

1. Mitnitski AB, Mogilner AJ, Rockwood K: **Accumulation of deficits as a proxy measure of aging**. *ScientificWorldJournal* 2001, **1**:323-336.

2. Steverink N, Lindenberg S: **Which social needs are important for subjective well-being? What happens to them with aging?** *Psychol Aging* 2006, **21**(2):281-290.

3. Andrew MK, Keefe JM: **Social vulnerability from a social ecology perspective: a cohort study of older adults from the National Population Health Survey of Canada**. *BMC Geriatr* 2014, **14**:90.

4. Bunt S, Steverink N, Olthof J, Schans C, Hobbelen J: **Social frailty in older adults: a scoping review**. *European Journal of Ageing* 2017, **14**(3):323-334.

5. Pinsker DM, Stone V, Pachana N, Greenspan S: **Social Vulnerability Scale for older adults: Validation study**. *Clinical Psychologist* 2006, **10**(3):109-119.

6. Pinsker DM, McFarland K, Stone VE: **The Social Vulnerability Scale for Older Adults: An Exploratory and Confirmatory Factor Analytic Study**. *J Elder Abuse Negl* 2011, **23**(3):246-272.

7. Pinsker DM: **Clinical application of the Social Vulnerability Scale: Three illustrative case reports of patients with dementia**. *Clinical Psychologist* 2011, **15**(1):33-43.

8. Andrew MK, Mitnitski AB, Rockwood K: **Social vulnerability, frailty and mortality in elderly people**. *PLoS One* 2008, **3**(5):e2232.

9. Garre-Olmo J, Calvo-Perxas L, Lopez-Pousa S, de Gracia Blanco M, Vilalta-Franch J: **Prevalence of frailty phenotypes and risk of mortality in a community-dwelling elderly cohort**. *Age Ageing* 2013, **42**(1):46-51.

10. Makizako H, Shimada H, Tsutsumimoto K, Lee S, Doi T, Nakakubo S, Hotta R, Suzuki T: **Social Frailty in Community-Dwelling Older Adults as a Risk Factor for Disability**. *J Am Med Dir Assoc* 2015, **16**(11):1003 e1007-1011.

11. Teo N, Gao Q, Nyunt MSZ, Wee SL, Ng T-P: **Social Frailty and Functional Disability: Findings From the Singapore Longitudinal Ageing Studies**. *Journal of the American Medical Directors Association* 2017, **18**(7):637.e613-637.e619.

12. Ma L, Sun F, Tang Z: **Social Frailty Is Associated with Physical Functioning, Cognition, and Depression, and Predicts Mortality**. *J Nutr Health Aging* 2018, **22**(8):989-995.

13. Yamada M, Arai H: **Social Frailty Predicts Incident Disability and Mortality Among Community-Dwelling Japanese Older Adults**. *J Am Med Dir Assoc* 2018, **19**(12):1099-1103.

14. Yoo M, Kim S, Kim BS, Yoo J, Lee S, Jang HC, Cho BL, Son SJ, Lee JH, Park YS *et al*: **Moderate hearing loss is related with social frailty in a community-dwelling older adults: The Korean Frailty and Aging Cohort Study (KFACS)**. *Arch Gerontol Geriatr* 2019, **83**:126-130.

15. Pek K, Chew J, Lim JP, Yew S, Tan CN, Yeo A, Ding YY, Lim WS: **Social frailty is independently associated with mood, nutrition, physical performance, and physical activity: Insights from a theory-guided approach**. *International Journal of Environmental Research and Public Health* 2020, **17**(12):1-15.

16. Chen Z, Jiang X, Shi G, Wang Y, Chu X, Wang Z, Guo J, Zhu Y, Chen J, Wang X *et al*: **Social frailty and longitudinal risk of depressive symptoms in a Chinese population: the Rugao Longevity and Aging Study**. *Psychogeriatrics* 2021, **21**(4):483-490.

17. Irshad C, Govil D, Sahoo H: **Social frailty among older adults in India: Findings from the Longitudinal Ageing Study in India (LASI) - Wave 1**. *Experimental aging research* 2024, **50**(3):331-347.

18. Inoue T, Maeda K, Satake S, Matsui Y, Arai H: **Osteosarcopenia, the co-existence of osteoporosis and sarcopenia, is associated with social frailty in older adults**. *Aging Clin Exp Res* 2022, **34**(3):535-543.

19. Nagai K, Tamaki K, Kusunoki H, Wada Y, Tsuji S, Itoh M, Sano K, Amano M, Hayashitani S, Yokoyama R *et al*: **Physical frailty predicts the development of social frailty: a prospective cohort study**. *BMC Geriatr* 2020, **20**(1):403.

20. Shega JW, Andrew M, Hemmerich J, Cagney KA, Ersek M, Weiner DK, Dale W: **The Relationship of Pain and Cognitive Impairment with Social Vulnerability-An Analysis of the Canadian Study of Health and Aging**. *Pain Medicine* 2012, **13**(2):190-197.

21. Kodama A, Kume Y, Lee S, Makizako H, Shimada H, Takahashi T, Ono T, Ota H: **Impact of COVID-19 Pandemic Exacerbation of Depressive Symptoms for Social Frailty from the ORANGE Registry**. *International Journal of Environmental Research and Public Health* 2022, **19**(2).

22. Kume Y, Kodama A, Takahashi T, Lee S, Makizako H, Ono T, Shimada H, Ota H: **Social frailty is independently associated with geriatric depression among older adults living in northern Japan: A cross‐sectional study of ORANGE registry**. *Geriatrics & Gerontology International* 2022, **22**(2):145-151.

23. van Oostrom SH, van der AD, Rietman ML, Picavet HSJ, Lette M, Verschuren WMM, de Bruin SR, Spijkerman AMW: **A four-domain approach of frailty explored in the Doetinchem Cohort Study**. *BMC Geriatr* 2017, **17**(1):196.

24. Ye L, Elstgeest LEM, Zhang X, Alhambra-Borrás T, Tan SS, Raat H: **Factors associated with physical, psychological and social frailty among community-dwelling older persons in Europe: a cross-sectional study of Urban Health Centres Europe (UHCE)**. *BMC Geriatr* 2021, **21**(1):422.

25. Nakakubo S, Doi T, Makizako H, Tsutsumimoto K, Kurita S, Kim M, Ishii H, Suzuki T, Shimada H: **Association of sleep condition and social frailty in community‐dwelling older people**. *Geriatrics & Gerontology International* 2019, **19**(9):885-889.

26. Henry JD, Coundouris SP, Mead J, Thompson B, Hubbard RE, Grainger SA: **Social Frailty in Late Adulthood: Social Cognitive and Psychological Well-Being Correlates**. *J Gerontol B Psychol Sci Soc Sci* 2023, **78**(1):87-96.

27. Hirase T, Makizako H, Okubo Y, Lord SR, Inokuchi S, Okita M: **Chronic pain is independently associated with social frailty in community‐dwelling older adults**. *Geriatrics & Gerontology International* 2019, **19**(11):1153-1156.

28. Gobbens RJJ, Kuiper S, Dijkshoorn H, van Assen MALM: **Associations of individual chronic diseases and multimorbidity with multidimensional frailty**. *Archives of Gerontology and Geriatrics* 2024, **117**.

29. Park H, Jang IY, Lee HY, Jung HW, Lee E, Kim DH: **Screening value of social frailty and its association with physical frailty and disability in community-dwelling older Koreans: Aging study of pyeongchang rural area**. *International Journal of Environmental Research and Public Health* 2019, **16**(16).

30. Doi T, Tsutsumimoto K, Ishii H, Nakakubo S, Kurita S, Kiuchi Y, Nishimoto K, Shimada H: **Impact of social frailty on the association between driving status and disability in older adults**. *Arch Gerontol Geriatr* 2022, **99**:104597.

31. Doi T, Tsutsumimoto K, Makino K, Nakakubo S, Sakimoto F, Matsuda S, Shimada H: **Combined Social Frailty and Life-Space Activities Associated with Risk of Disability: A Prospective Cohort Study**. *Journal of Frailty and Aging* 2024.

32. Cooper F, Lewis EG, Urasa S, Whitton L, Collin H, Coles S, Wood GK, Ali AM, Mdegella D, Mkodo J *et al*: **Social Vulnerability, Frailty, and Their Association With Mortality in Older Adults Living in Rural Tanzania**. *The journals of gerontology Series A, Biological sciences and medical sciences* 2022, **77**(10):2050-2058.

33. Sun QQ, Tan K, Tang HY, Liu YY, Zhu H, Qin H, Xia X, Zhang M, Chen YY, Nie SS *et al*: **Incidence and predictive value of social frailty among community-dwelling older adults in Southwest China: A prospective cohort study**. *Frontiers in public health* 2023, **11**:1103651.

34. Makizako H, Shimada H, Doi T, Tsutsumimoto K, Hotta R, Nakakubo S, Makino K, Lee S: **Social frailty leads to the development of physical frailty among physically non-frail adults: A four-year follow-up longitudinal cohort study**. *International Journal of Environmental Research and Public Health* 2018, **15**(3).

35. Makizako H, Kubozono T, Kiyama R, Takenaka T, Kuwahata S, Tabira T, Kanoya T, Horinouchi K, Shimada H, Ohishi M: **Associations of social frailty with loss of muscle mass and muscle weakness among community‐dwelling older adults**. *Geriatrics & Gerontology International* 2019, **19**(1):76-80.

36. Huang CH, Okada K, Matsushita E, Uno C, Satake S, Martins BA, Kuzuya M: **The association of social frailty with intrinsic capacity in community-dwelling older adults: a prospective cohort study**. *BMC Geriatr* 2021, **21**(1):515.

37. Armstrong JJ, Mitnitski A, Andrew MK, Launer LJ, White LR, Rockwood K: **Cumulative impact of health deficits, social vulnerabilities, and protective factors on cognitive dynamics in late life: A multistate modeling approach**. *Alzheimer's Research and Therapy* 2015, **7**(1).

38. Zhang H, Hu Z, Jiang S, Hao M, Li Y, Liu Y, Jiang XY, Jin L, Wang X: **Social frailty and the incidence of motoric cognitive risk syndrome in older adults**. *Alzheimer's and Dementia* 2024, **20**(4):2329-2339.

39. Hayashi T, Noguchi T, Kubo Y, Tomiyama N, Ochi A, Hayashi H: **Social frailty and depressive symptoms during the COVID-19 pandemic among older adults in Japan: Role of home exercise habits**. *Arch Gerontol Geriatr* 2022, **98**:104555.

40. Ko H, Jung S: **Association of social frailty with physical health, cognitive function, psychological health, and life satisfaction in community-dwelling older Koreans**. *International Journal of Environmental Research and Public Health* 2021, **18**(2):1-9.

41. Noguchi T, Nojima I, Inoue-Hirakawa T, Sugiura H: **Association between Social Frailty and Sleep Quality among Community-dwelling Older Adults: A Cross-sectional Study**. *Phys Ther Res* 2021, **24**(2):153-162.

42. Pollak C, Wexler SS, Drury L: **Effect of a Robotic Pet on Social and Physical Frailty in Community-Dwelling Older Adults: A Randomized Controlled Trial**. *Res Gerontol Nurs* 2022, **15**(5):229-237.

43. Hilt AJ, Javedan H: **The impact of social vulnerability on readmission of hospitalized older adults**. *Journal of the American Geriatrics Society* 2018, **66**:S110-S111.

44. Lina Ma, Zhe Tang, Fei Sun, Li Y: **Development of a tool for identifying social frailty in Beijing**

**Longitudinal Study of Aging (P-676), Abstracts of the 15th International Congress of the European Geriatric Medicine Society**. *European Geriatric Medicine* 2019, **10**(1):1-325.

45. Osokpo OH, Iroegbu C, Huang L, Lewis LM, Hirschman K, Naylor MD, Riegel BJ: **Higher Social Vulnerability is Associated With Lower Self-Care Monitoring in African Immigrants With Chronic Disease**. *Circulation* 2022, **146**.

46. Shahid I, Alias A, Nwana N, Gullapelli R, Khan S, Kundi H, Hagan K, Nicolas J, Bose B, Javed Z *et al*: **SOCIAL VULNERABILITY INDEX AS A PREDICTOR OF MAJOR ADVERSE CARDIOVASCULAR EVENTS AMONG PATIENTS IN A LARGE INTEGRATED HEALTHCARE SYSTEM: HOUSTON METHODIST CVD LEARNING HEALTH SYSTEM REGISTRY**. *Journal of the American College of Cardiology* 2024, **83**(13):1340.

47. Lim W, Pek K, Lim J, Chew J: **Social Frailty and the Covid-19 Pandemic: Disentangling the Bi-Directional Relationship**. *Journal of the American Geriatrics Society* 2023, **71**:S160.

48. Fowler ME, Harmon C, Tucker A, Sharafeldin N, Giri S, Bhatia S, Williams G: **The Association of Social Vulnerability with Geriatric Assessment Impairments among Older Adults with Gastrointestinal Cancers- The CARE Registry**. *Journal of the American Geriatrics Society* 2023, **71**:S152.

49. Jain V, Al Rifai M, Khan SU, Kalra A, Rodriguez F, Samad Z, Pokharel Y, Misra A, Sperling LS, Rana JS *et al*: **Association Between Social Vulnerability Index and Cardiovascular Disease: A Behavioral Risk Factor Surveillance System Study**. *J Am Heart Assoc* 2022, **11**(15):e024414.

50. Neiman PU, Flaherty MM, Salim A, Sangji NF, Ibrahim A, Fan Z, Hemmila MR, Scott JW: **Evaluating the complex association between Social Vulnerability Index and trauma mortality**. *J Trauma Acute Care Surg* 2022, **92**(5):821-830.

51. Miao W, Zhang X, Shi B, Tian W, Wu B, Lai Y, Li Y, Huang Z, Xia Q, Yang H *et al*: **Multi-dimensional vulnerability analysis on catastrophic health expenditure among middle-aged and older adults with chronic diseases in China**. *BMC Medical Research Methodology* 2022, **22**(1):151.

52. Wang J, Xu S, Liu J, Yan Z, Zhang S, Liu M, Wang X, Wang Z, Liang Q, Luan X: **The mediating effects of social support and depressive symptoms on activities of daily living and social frailty in older patients with chronic heart failure**. *Geriatric Nursing* 2023, **53**:301-306.

53. Chen KY, Blackford AL, Sedhom R, Gupta A, Hussaini SMQ: **Local Social Vulnerability as a Predictor for Cancer-Related Mortality Among US Counties**. *Oncologist* 2023, **28**(9):e835-e838.

54. Pascal J, Abbey-Huguenin H, Agard C, Asseray N, Billaud É, Baron D, Lombrail P: **Development of a tool for the identification of socially vulnerable hospital outpatients**. *Presse Medicale* 2004, **33**(11):710-715.

55. Díaz-Alonso J, Bueno-Pérez A, Toraño-Ladero L, Caballero FF, López-García E, Rodríguez-Artalejo F, Lana A: **Hearing loss and social frailty in older men and women**. *Gaceta sanitaria* 2021, **35**(5):425-431.

56. dos Santos IL, Zimmermann IR, Donalísio MR, Santimaria MR, Sanchez MN, de Carvalho JLB, Borim FSA: **Social vulnerability, survival, and hospital lethality by COVID-19 in patients aged 50 years and over: retrospective cohort of cases in Brazil in 2020 and 2021**. *Cadernos de Saude Publica* 2022, **38**(11).

57. Nagano M, Kabayama M, Ohata Y, Rakugi H, Kamide K: **The usefulness of a questionnaire during medical examinations for older subjects in evaluating frailty: Utilization in clinical practice**. *Nihon Ronen Igakkai zasshi Japanese journal of geriatrics* 2022, **59**(3):360-370.

58. Tarazona-Santabalbina FJ, Gómez-Cabrera MC, Pérez-Ros P, Martínez-Arnau FM, Cabo H, Tsaparas K, Salvador-Pascual A, Rodriguez-Mañas L, Viña J: **A Multicomponent Exercise Intervention that Reverses Frailty and Improves Cognition, Emotion, and Social Networking in the Community-Dwelling Frail Elderly: A Randomized Clinical Trial**. *J Am Med Dir Assoc* 2016, **17**(5):426-433.

59. Verver D, Merten H, de Blok C, Wagner C: **A cross sectional study on the different domains of frailty for independent living older adults**. *BMC Geriatr* 2019, **19**(1):61.

60. Aznar-Tortonda V, Palazón-Bru A, la Rosa DMF, Espínola-Morel V, Pérez-Pérez BF, León-Ruiz AB, Gil-Guillén VF: **Detection of frailty in older patients using a mobile app: cross-sectional observational study in primary care**. *Br J Gen Pract* 2020, **70**(690):e29-e35.

61. Hoogendijk EO, Smit AP, van Dam C, Schuster NA, de Breij S, Holwerda TJ, Huisman M, Dent E, Andrew MK: **Frailty Combined with Loneliness or Social Isolation: An Elevated Risk for Mortality in Later Life**. *J Am Geriatr Soc* 2020, **68**(11):2587-2593.

62. Gelmini G, Pettenati P, Baratta S, Loss MG, Lunghi M, Veronese N: **Evaluation of bio-psycho-social frailty in older persons on the territory: the method and the experience of the "Medesano Health House"**. *Acta Biomed* 2020, **91**(2):389-395.

63. Harada K, Masumoto K, Katagiri K, Fukuzawa A, Touyama M, Sonoda D, Chogahara M, Kondo N, Okada S: **Three-year effects of neighborhood social network intervention on mental and physical health of older adults**. *Aging Ment Health* 2021, **25**(12):2235-2245.

64. De Luca R, Torrisi M, Bramanti A, Maggio MG, Anchesi S, Andaloro A, Caliri S, De Cola MC, Calabrò RS: **A multidisciplinary Telehealth approach for community dwelling older adults**. *Geriatr Nurs* 2021, **42**(3):635-642.

65. Papathanasiou IV, Fradelos EC, Mantzaris D, Rammogianni A, Malli F, Papagiannis D, Gourgoulianis KI: **Multimorbidity, Trauma Exposure, and Frailty of Older Adults in the Community**. *Frontiers in Genetics* 2021, **Volume 12 - 2021**.

66. Melchiorre MG, D'Amen B, Quattrini S, Lamura G, Socci M: **Health Emergencies, Falls, and Use of Communication Technologies by Older People with Functional and Social Frailty: Ageing in Place in Deprived Areas of Italy**. *Int J Environ Res Public Health* 2022, **19**(22).

67. Roth SE, Govier DJ, Marsi K, Cohen-Cline H: **Differences in Outpatient Health Care Utilization 12 Months after COVID-19 Infection by Race/Ethnicity and Community Social Vulnerability**. *Int J Environ Res Public Health* 2022, **19**(6).

68. Millan-Domingo F, Tarazona-Santabalbina FJ, Carretero A, Olaso-Gonzalez G, Vina J, Gomez-Cabrera MC: **Real-Life Outcomes of a Multicomponent Exercise Intervention in Community-Dwelling Frail Older Adults and Its Association with Nutritional-Related Factors**. *Nutrients* 2022, **14**(23).

69. Satsanasupint P, Daovisan H, Phukrongpet P: **Enhancing active ageing in later life: Can community networks enhance elderly health behaviours? Insights from a bracketing qualitative method**. *Journal of Community & Applied Social Psychology* 2022, **32**(6):1133-1147.

70. Faria A, Martins M, Ribeiro O, Ventura-Silva JMA, Fonseca EF, Ferreira LJM, Teles P, Laredo-Aguilera JA: **Multidimensional Frailty and Lifestyles of Community-Dwelling Older Portuguese Adults**. *Int J Environ Res Public Health* 2022, **19**(22).

71. Jacobs MM, Burch AE: **Disparities in Perceived Physical and Mental Wellness: Relationships Between Social Vulnerability, Cardiovascular Risk Factor Prevalence, and Health Behaviors Among Elderly US Residents**. *J Prim Care Community Health* 2023, **14**:21501319231163639.

72. Ohta T, Osuka Y, Shida T, Daimaru K, Kojima N, Maruo K, Iizuka A, Kitago M, Fujiwara Y, Sasai H: **Feasibility, Acceptability, and Potential Efficacy of a Mobile Health Application for Community-Dwelling Older Adults with Frailty and Pre-Frailty: A Pilot Study**. *Nutrients* 2024, **16**(8):1181.
